# The *Aedes aegypti* Toll Pathway Controls Dengue Virus Infection

**DOI:** 10.1371/journal.ppat.1000098

**Published:** 2008-07-04

**Authors:** Zhiyong Xi, Jose L. Ramirez, George Dimopoulos

**Affiliations:** W. Harry Feinstone Department of Molecular Microbiology and Immunology, Bloomberg School of Public Health, Johns Hopkins University, Baltimore, Maryland, United States of America; Stanford University, United States of America

## Abstract

*Aedes aegypti*, the mosquito vector of dengue viruses, utilizes its innate immune system to ward off a variety of pathogens, some of which can cause disease in humans. To date, the features of insects' innate immune defenses against viruses have mainly been studied in the fruit fly *Drosophila melanogaster*, which appears to utilize different immune pathways against different types of viruses, in addition to an RNA interference–based defense system. We have used the recently released whole-genome sequence of the *Ae. aegypti* mosquito, in combination with high-throughput gene expression and RNA interference (RNAi)-based reverse genetic analyses, to characterize its response to dengue virus infection in different body compartments. We have further addressed the impact of the mosquito's endogenous microbial flora on virus infection. Our findings indicate a significant role for the Toll pathway in regulating resistance to dengue virus, as indicated by an infection-responsive regulation and functional assessment of several Toll pathway–associated genes. We have also shown that the mosquito's natural microbiota play a role in modulating the dengue virus infection, possibly through basal-level stimulation of the Toll immune pathway.

## Author Summary

The *Aedes aegypti* mosquito is largely responsible for the transmission of dengue viruses that cause disease in humans. The virus is taken up with an infected blood meal from which it will first infect the mosquito gut tissue. From the gut it will migrate to other parts of the mosquito, including the salivary glands, from where it can be transmitted to another human upon a second blood meal. In this study we show that the mosquito utilizes its innate immune system to control dengue virus infection. Infection with the virus will result in the activation of mosquito immune responses that are mainly controlled by the Toll pathway. These responses entail antiviral activities that limit infection with the virus. We also show that the mosquito's natural microbial flora play a role in modulating the dengue virus infection, possibly through the stimulation of the mosquito's immune system.

## Introduction

The dengue viruses, whose geographic distribution resembles that of malaria, has become the most important arboviral pathogen in recent years because of its increasing incidence in the tropics and subtropics as well as its high morbidity and mortality. The public health impact of dengue is enormous, given that 2.5 billion people live in dengue-endemic areas and are at daily risk of infection [1]. The dengue viruses are single-stranded positive RNA belonging to the family *Flaviviridae*, genus *Flavivirus*. They are transmitted between humans primarily by the mosquito *Ae. aegypti* and by *Ae. albopictus* as a secondary vector [2]. The four closely related dengue serotypes are antigenically distinct, each comprising several genotypes that exhibit differences in their infection characteristics in both the mosquito vector and the human host [3],[4].

The extrinsic incubation period of dengue viruses in the mosquito is 7–14 days and is dependent on the mosquito strain, virus genotype, and environmental factors such as humidity and temperature [5],[6]. When the mosquito ingests a dengue-infected blood meal, the virus first infects the midgut tissue, within which it replicates to produce more virus particles. It then spreads through the hemolymph to other tissues such as the trachea, fat body, and salivary glands, where it is further propagated through replication. Peak virus titers usually occur between 7 and 10 days post-infection in the midgut and between 7 and 17 days in the abdomen. Peak levels in the head and salivary gland occur later, at about 12–18 days after feeding [7]. This extrinsic incubation time varies for different virus-vector combinations, and the tropism of the virus is dependent on the mosquito's tissue- and cell-specific susceptibility to different genotypes [5],[7].

In arthropods, innate immunity plays an important role in limiting pathogen infection, both through the production of effector molecules such as antimicrobial peptides and through phagocytosis and encapsulation, secretion of physical barriers, and melanization [8]. Studies that were mainly conducted in the insect model *D. melanogaster* have shown that arthropod immune responses are largely regulated by two main pathways, the Toll and immune deficiency (Imd) pathways [9],[10].

Activation of the Toll pathway by microbes through pattern recognition receptors (PRRs) leads to a cascade of events that result in the degradation of the negative regulator Cactus, translocation to the nucleus of transcription factors such as Dif, and a rapid increase in antimicrobial compounds and other effectors [10]–[12]. The Imd pathway is involved in the defense against Gram-negative bacteria, and upon activation it follows a cascade of events similar to those in the Toll pathway, involving putative degradation of its negative regulator Caspar, translocation of the transcription factor Relish to the nucleus, and the production of effectors and antimicrobial compounds [13],[14]. In contrast to the relatively well-characterized Toll and Imd pathways, less is known about the Janus kinase signal transducers and activators of transcription (JAK/STAT) pathway, which comprises multiple factors and has been linked to immune responses in the fruit fly [15],^16^. Comparative genomics analyses have shown a striking degree of conservation of these immune signaling pathways in *D. melanogaster*, *Anopheles gambiae* and *Ae. aegypti*; in contrast, the upstream pattern recognition receptors and the downstream effectors have differentiated quite significantly among the three species, probably as a result of different microbial exposures [17].

The Rel family transcription factors, Dif and Relish in Drosophila or their corresponding Rel1 and Rel2 in mosquitoes, can be studied through RNA interference (RNAi)-mediated silencing of the negative regulators Cactus and Caspar, respectively [11],[13],[14]. This approach allows a transient simulation, to at least a partial degree, of the Toll and Imd pathways in the absence of a microbial elicitor. The activation of these pathways can be monitored through the transcriptional activation of some of the signal cascade factors, such as the up-regulation of the Rel family transcription factors and down-regulation of the negative regulator Cactus or Caspar for the Toll or Imd pathway, respectively [11],[13],[14].

At present, relatively little is known about the anti-viral defense systems in insects. In *D. melanogaster*, the RNAi-mediated defenses appear to be key players in the defense against a broad range of viruses [18],[19], while some of the classical innate immune pathways such as the Toll and JAK-STAT pathways have also been implicated in limiting virus infection [20],[21]. Specifically, *D. melanogaster* has been shown to use its RNAi machinery and the Toll pathway to limit *Drosophila*×virus infection (a member of the *Dicistroviridae*) [19],[21], and it uses its RNAi machinery and the JAK-Stat pathway to limit *Drosophila* C virus infection (a member of the *Birnavirus* family) [20],[22]. Another study has demonstrated the involvement of the *D. melanogaster* RNAi machinery in the defense against two diverse animal viruses: a flock house virus and a cricket paralysis virus [18]. With all the above knowledge, however, the molecular mechanisms that govern their activation after infection and their role in virus clearance are unknown. Links between the RNAi machinery and the innate immune signaling pathways have yet not been identified [18],[23].

Similarly, limited knowledge on the antiviral response in mosquitoes is available. In *Ae. aegypti*, Sindbis virus (*Alphavirus*; *Togaviridae*) infection has been shown to induce the Toll pathway-related Rel1 transcription factor and three transcripts of the ubiquitin-ligase pathway genes, which are known regulators of NFkB-like proteins [24]. The RNAi machinery has also been linked to the anti-dengue defense in *Ae. aegypti* [25] and anti- O'nyong-nyong virus (*Alphavirus; Togaviridae*) in *An. gambiae* [26]. In addition, the O'nyong-nyong virus has been shown to induce 18 genes in *A. gambiae*, including a 70-kDa heat shock protein factor that later was shown to influence the virus's ability to propagate in the vector [27].

The recently available *Ae. aegypti* genome sequence [28], in combination with high-throughput gene expression and reverse genetic methodology, have provided unprecedented opportunities to study the mosquito's responses and defenses against dengue virus infection. Here, we report the global transcriptional response of *Ae. aegypti* to the infection of dengue virus serotype 2 (DENV-2), and show that DENV-2 induces a set of genes corresponding to the Toll and JAK-STAT pathways. Activation of the Toll and Imd pathways in *Ae. aegypti* through RNAi-mediated silencing of Cactus and Caspar caused a reduction in dengue virus infection level that appeared to be controlled primarily by the Toll pathway. Repression of the Toll pathway through MYD88 gene silencing resulted in higher dengue virus infection levels. We also present compelling evidence for an inhibitory effect of the mosquito's natural microbiota on virus infection and discuss the implications of these findings and the potential role of the mosquito's microbial exposure and innate immune system in modulating dengue virus transmission.

## Results

### Global transcriptome responses to dengue infection at 10 days after an infected blood meal

**Figure 1:**
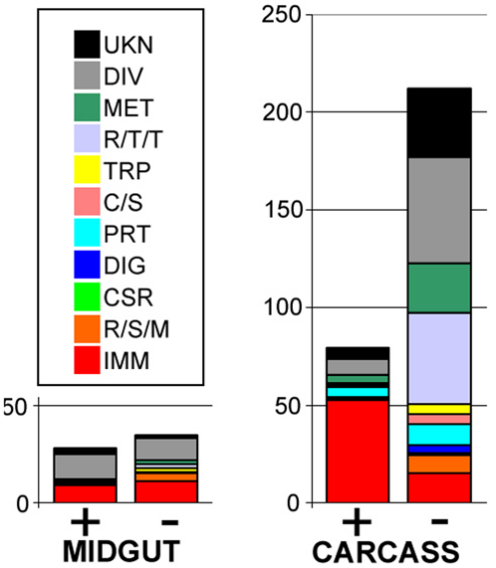
Functional classification of differentially expressed genes in the dengue-infected midgut and carcass at 10 days after blood meal. The graph shows the functional class distributions in real numbers of genes that are regulated by virus infection (+ indicate induced and – indicate repressed). The virus infection responsive gene expression data are presented in Tables S1 and S2. Functional group abbreviations: IMM, immunity; R/S/M, redox, stress and mitochondrion; CSR, chemosensory reception; DIG, blood and sugar food digestive; PRT, proteolysis; C/S, cytoskeletal and structural; TRP, transport; R/T/T, replication, transcription, and translation; MET, metabolism; DIV, diverse functions; UNK, unknown functions.

We first assessed the physiological response of the *Ae. aegypti* mosquito to systemic dengue infection at the gene-specific level in the midgut and remaining carcass by using a genome-wide transcriptional profiling approach. A comparison of the transcript abundance in the two body compartments of mosquitoes that were fed 10 days earlier on dengue-infected blood or naïve blood revealed broad responses to virus infection that entailed a variety of physiological systems (Fig 1). The carcass displayed a significantly larger number of regulated genes (240 up-regulated and 192 down-regulated) than did the smaller midgut tissue (28 up-regulated and 35 down-regulated). The magnitude of the gene regulation, as measured by the -fold change in transcript abundance, was also greater in the carcass, suggesting that tissues in the carcass are at this stage of infection more actively engaged in the response to infection, while the midgut tissue may have reached a steady-state/balance in its interaction with the virus (Tables S1 and S2). A fairly large proportion (33.5%) of the genes displayed a similar expression profile in the midgut and the carcass (Tables 1, S1, and S2). The most striking infection-responsive gene regulation was observed for genes with putative functions related to the mosquito's innate immune system; these genes represented 34.5% in the midgut and 27.5% in the carcass of all the regulated genes with predicted functions (Fig. 1). Other major functional gene groups that were affected by virus infection included metabolism, oxidoreductive processes, and stress responsive systems, and are discussed in greater detail in Text S1.

**Table 1:** Differentially expressed putative immune genes in the dengue-infected midgut and carcass and their overlap with those of Cactus- and Caspar-silenced mosquitoes.

Gene ID	Gene Name	No	Function group	Logfold			
				Carcass	Midgut	dsCact	dsCaspar
AAEL000709	CACT	62	Toll	−0.842	−0.084	0.515	0.074
AAEL007696	REL1A	64	Toll	0.924	−0.096	1.005	0.157
AAEL001929	SPZ5	63	Toll	1.61	0.037	0.034	0.106
AAEL003507	TOLL1B	66	Toll		0.947	0.014	0.08
AAEL013441	TOLL9A	65	Toll	1.189	−0.036	−0.054	0.149
AAEL004223	CECB	5	Effector	0.544	0.81	−0.148	0.61
AAEL015515	CECG	6	Effector	1.052	0.131	1.394	−2.992
AAEL003832	DEFC	9	Effecttor	−1.81	0.143	0.95	−1.665
AAEL003857	DEFD	8	Effector	−0.127	1.076	0.999	−1.962
AAEL003849	DEFE	7	Effector	0.824	0.053	−0.811	1.697
AAEL004522	GAM	10	Effector	0.851	1.118	−1.406	0.85
AAEL015404	LYSC	11	Effector	1.007	0.935	1.105	0.082
AAEL006702	FREP	31	Pattern Recognition Receptor	1.143	0.031	−0.333	−0.009
AAEL006699	FREP	32	Pattern Recognition Receptor		−1.129	−1.297	0.016
AAEL006704	FREP	33	Pattern Recognition Receptor	0.073	−0.896	−1.128	0.313
AAEL000652	GNBPA2	28	Pattern Recognition Receptor	0.805	0.928	0.041	−0.025
AAEL009178	GNBPB4	30	Pattern Recognition Receptor	0.92	−0.126	−0.065	0.061
AAEL007064	GNBPB6	29	Pattern Recognition Receptor	0.886	0.088	0.118	−1.077
AAEL003325	ML	34	Pattern Recognition Receptor	−0.949	0.85	0.05	0.083
AAEL009531	ML	35	Pattern Recognition Receptor	1.427	−0.072	0.031	−0.83
AAEL006854	ML	36	Pattern Recognition Receptor	0.031	1.143	0.263	0.219
AAEL014989	PGPPLD, putative	38	Pattern Recognition Receptor	2.11	0.101	−0.155	−0.113
AAEL011608	PGRPLD	37	Pattern Recognition Receptor	1.962	0.011	−0.098	−0.099
AAEL012267	TEP13	41	Pattern Recognition Receptor	1.325	0.084	0.112	0.8
AAEL014755	TEP15	42	Pattern Recognition Receptor	1.19	−0.023	1.628	0.168
AAEL001794	TEP20	40	Pattern Recognition Receptor	0.896	0.191	1.518	0.313
AAEL000087	TEP22	39	Pattern Recognition Receptor	1.819	0.084	1.896	0.317
Aaeg:N19306	TEP24	44	Pattern Recognition Receptor	0.8943			
Aaeg:N18111	TEP25	43	Pattern Recognition Receptor	1.2427			
AAEL003253	CLIPB13B	45	Signal Modulation	1.038	0.209	1.638	0.003
AAEL005093	CLIPB46	48	Signal Modulation		−0.913	1.121	0.344
AAEL005064	CLIPB5	46	Signal Modulation	−0.852	0.059	1.548	0.315
AAEL007593	CLIPC2	47	Signal Modulation	−0.815	0.15	1.379	0.155
AAEL014390	CTL	52	Signal Modulation	0.986	0.162	0.942	0.188
AAEL003119	CTL6	49	Signal Modulation	0.85	0.018	0.12	−0.009
AAEL011619	CTLGA8	51	Signal Modulation	0.986	0.085	1.129	0.281
AAEL011455	CTLMA12	50	Signal Modulation	1.095	0.134	2.473	0.216
AAEL000256	SCRB9	53	Signal Modulation	1.036	0.203	0.223	0.023
AAEL014079	SRPN1	59	Signal Modulation	0.915	−0.017	0.995	−0.027
AAEL007765	SRPN10A	61	Signal Modulation	0.166	−0.963	0.841	−0.009
AAEL014078	SRPN2	58	Signal Modulation	0.884	−0.048		−2.026
AAEL002730	SRPN21	54	Signal Modulation	1.426	0.128	0.41	0.148
AAEL002715	SRPN22	60	Signal Modulation	0.12	1.244	0.062	0.166
AAEL013936	SRPN4A	57	Signal Modulation	1.35	0.041	1.426	0.156
AAEL013934	SRPN4D	56	Signal Modulation	1.343	0.217	0.91	0.259
AAEL008364	SRPN9	55	Signal Modulation	−0.951	−0.031	1.275	0.169
AAEL000393	Suppressors of cytokine signalling	13	JAK-STAT	0.909	0.058	0.186	0.103
AAEL009645	Hypothetical protein	14	JAK-STAT	−0.846	−0.584	0.427	−0.012
AAEL009822	Metabotropic glutamate receptor	15	JAK-STAT	1.405	0.185	−0.086	0.028
AAEL012471	DOME	16	JAK-STAT	1.078	−0.06	1.561	−0.868
AAEL012510	IKK2	12	Imd	−0.912	−1.042	0.043	−0.034
AAEL003439	CASPS18	1	Apoptosis	0.803	0.017	−0.821	−0.094
AAEL012143	CASPS7	2	Apoptosis	−0.854	0.014	−0.068	−0.034
AAEL011562	CASPL2	3	Apoptosis	−0.606	−0.839	0.183	0.076
AAEL014658	CASPS20	4	Apoptosis		−0.898	−0.064	0.019
Aaeg:N41501	CAT1A	17	Oxidative defense enzymes		−0.84617		
AAEL004386	HPX8C	18	Oxidative defense enzymes	−1.106	−0.031	−1.745	0.049
AAEL004388	HPX8A	19	Oxidative defense enzymes	−1.685	0.047	−2.054	0.094
AAEL004390	HPX8B	20	Oxidative defense enzymes	−1.034	0.063	−1.357	0.268
AAEL000274	CuSOD3, putative	21	Oxidative defense enzymes	−0.911	−0.119	−1.184	0.078
AAEL006271	CuSOD2	22	Oxidative defense enzymes	−0.841	−0.006	−1.099	−0.001
AAEL009436	SOD-Cu-Zn	23	Oxidative defense enzymes	−0.955	−0.473	0.173	0.148
AAEL011498	CuSOD3	24	Oxidative defense enzymes	−0.9	−0.16	−1.205	0.079
AAEL004112	TPX2	25	Oxidative defense enzymes	−1.433	−0.265	−0.84	0.06
AAEL014548	TPX3	26	Oxidative defense enzymes	−0.893	0.021	−0.17	−0.041
AAEL002309	TPX4	27	Oxidative defense enzymes	−0.301	−1.486	0.13	0.153

Dengue-infected midguts and carcasses were dissected and collected from the mosquitoes at 10 day after the blood meal. Injection of dsRNA of Cactus and Caspar into mosquitoes was conducted at 2 days post-emergence, and samples were collected for microarray analysis at 4 days after injection.

### Immune responses to dengue infection

**Figure 2:**
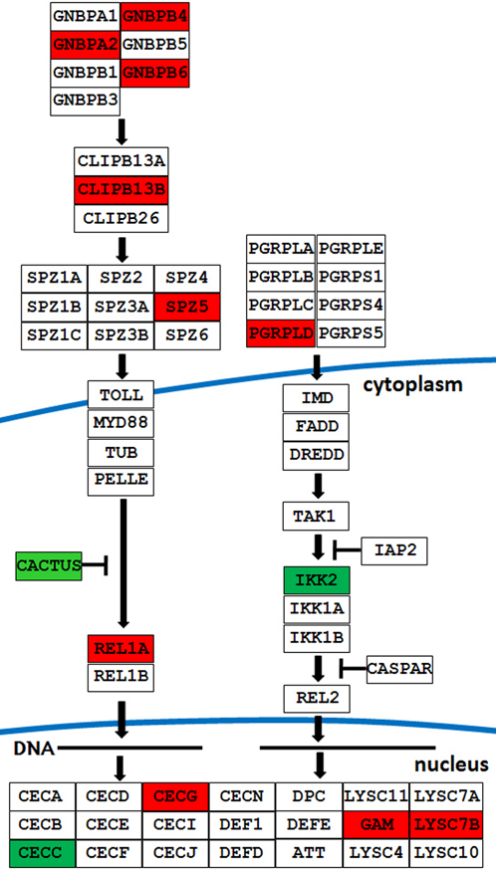
Regulation of putative Toll signaling pathway genes by dengue virus infection. Red color indicates infection responsive up-regulation and green color indicate infection responsive down-regulation. Non-colored gene boxes indicate lack of infection responsive regulation. The pathway was built with GenMapp software based on the immunogenomics prediction by Waterhouse *et al* 2007.

The 53 and 18 putative immune genes that were regulated by virus infection in the carcass and midgut tissues, respectively, were associated with a variety of immune functions such as PRRs, signaling modulation and transduction, effector systems, and apoptosis (Table 1). The functional group representations of the infection-responsive genes and their direction of regulation in the carcass and midgut tissues were quite similar, suggesting that the anti-viral responses involved the same types of defense mechanisms in these two compartments. For example, specific genes that displayed a similar pattern of regulation were lysozyme C (LYSC, AAEL015404), gambicin (AAEL004522), Ikkg (AAEL012510) and the Gram-negative binding protein A2 (GNBPA2, AAEL000652). A closer investigation of immune gene regulation using *in silico* comparative genomics analysis [17] revealed a striking bias toward genes putatively linked with the Toll immune signaling pathway (Fig. 2) as well as the JAK-STAT pathway. Activation of the Toll pathway in the carcass was supported by the up-regulation of Spaetzle (Spz), Toll, and Rel1A, and the down-regulation of the negative regulator Cactus. Three members of the Gram-negative bacteria-binding protein (GNBP) family were up-regulated, together with a clip-domain serine protease (CLIP), while the other two CLIPs were down-regulated; several antimicrobial effector molecules were up-regulated, including the defensins (DEFs), cecropins (CECs) and a lysozyme (LYSC). Only one predicted gene of the Imd immune signaling pathway, Ikkg, was down-regulated. One of the key components of the JAK-STAT pathway, Domeless (Dome), was induced upon dengue virus infection as well as three other genes (AAEL009645, AAEL009822 and AAEL000393) which have JAK-STAT pathway related orthologs in *D. melanogaster* [29]. Six members of the thio-ester containing protein (TEPs) gene family were also regulated by dengue infection, while TEP1 has been demonstrated to be a down-stream effector molecule of JAK-STAT pathway in *D. melanogaster* [30].

**Figure 3:**
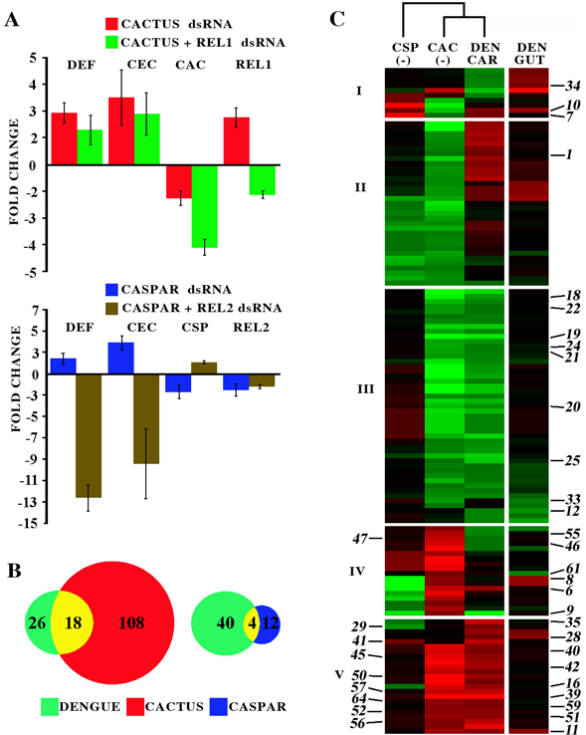
Comparative analysis of the dengue virus infection-responsive and Rel1 and Rel2 regulated transcriptomes. A. Expression analysis of defensin (DEF), cecropin (CEC), Cactus (CAC), and Rel1 in Cactus, and Cactus and Rel1 depleted mosquitoes (upper panel) and in Caspar, and Caspar and Rel2 depleted mosquitoes. Bar represents standard error. B. Venn diagram showing uniquely and commonly regulated genes in dengue infected and Cactus and Caspar depleted mosquitoes. C. Cluster analysis of 131 genes that were regulated in at least two of four treatments: dengue-infected midgut and carcass, and whole mosquitoes upon Cactus (CAC(-)) or Caspar (CSP(-)) depletion. The expression data of immune genes, indicated by the number beside the panel are presented in Table 1, and all genes presented in the hierarchical cluster matrix are listed in Table S6. The primary data for the real-time qPCR assays are presented in Table S3.

To establish further evidence that dengue infection activates the Toll immune signaling pathway, we designed experiments to assess the relationships between dengue infection-responsive gene regulation and Rel1- and Rel2-controlled gene regulation. Previous studies in *D. melanogaster* and *An. gambiae* have shown that the Rel1 and Rel2 transcription factors can be activated by depleting their negative regulators Cactus and Caspar, respectively [13],[14],[31]. To confirm that the Toll and Imd pathway had been activated, we depleted Cactus and Caspar using RNAi silencing and assayed the expression of the antimicrobial peptide genes DEF and CEC in gene-silenced mosquitoes and non-silenced controls (Fig. 3A). Gene silencing of either Cactus or Caspar induced the expression of these two genes. To link this activation to the Rel1 and Rel2 transcription factors, we performed double-knockdown assays in which both Cactus and Rel1 or Caspar and Rel2 were targeted simultaneously with RNAi and compared the effect of this double silencing on antimicrobial peptide gene expression to that of silencing the negative regulators alone. The double-knockdown treatments either compromised (in the case of Cactus and Rel1) or completely reversed (in the case of Caspar and Rel2) the effect induced by single-knockdown of Cactus or Caspar, respectively, indicating that these negative regulators could be used to activate these two transcription factors (Fig. 3A). The quantitative differences in the levels of de-activation of the Rel1- and Rel2-controlled transcription that were produced with this double-knockdown approach most likely reflect differences in the efficiency and kinetics of the RNAi-mediated depletion of different proteins.

We then determined the gene repertoires that were regulated by the Rel1 and Rel2 transcription factors, using a microarray-based approach in which we compared the transcript abundance in the Cactus and Caspar gene-silenced mosquitoes to that in GFP dsRNA-treated control mosquitoes. Our results indicated that differential gene regulation in the Cactus-depleted mosquitoes showed a strong bias toward the Toll pathway. For instance, we observed the up-regulation of Rel1 (AAEL007696), multiple Toll receptors (AAEL007619, AAEL000057, AAEL007613), Spätzle ligands (AAEL013434, AAEL008596), Gram-negative binding proteins (AAEL007626 and AAEL003889), and the antimicrobial peptides DEFD, CECA, D, E & G (AAEL003857, AAEL000627, AAEL000598, AAEL000611, AAEL015515). In total, Cactus gene silencing resulted in the up-regulation of 460 and down-regulation of 1423 genes belonging to different functional classes, with a predominant representation by immune genes (13.7% of all genes with predicted functions). The regulation of a variety of other functional gene groups by Rel1 is indicative of the multiple functional roles of the Toll pathway, including its contributions to immunity and development [32].

Differential gene regulation in Caspar-depleted mosquitoes was much less pronounced, with only 35 genes being induced and 137 being repressed. Those induced by Caspar silencing included TEP13 and the antimicrobial peptides DEFE and gambicin (AAEL004522 and AAEL003849). Rel1 and Rel2 are most likely regulating additional genes that were not detected because of the limited sensitivity of microarray-based gene expression assays.

A comparison of the dengue infection-responsive gene repertoire to that of Cactus gene-silenced mosquitoes showed a significant overlap, with 41% (18 of 44) of the immune genes being up-regulated by both the virus infection and Cactus gene silencing (Fig. 3B). In contrast, only 9% (4 of 44) of the dengue-regulated immune genes were also regulated in Caspar gene-silenced mosquitoes (Fig. 3B). Hierarchical clustering of genes that were differentially expressed in at least two of the three situations (Cactus silencing, Caspar silencing, and dengue infection) revealed a close relationship between Cactus silencing- and dengue infection-related regulation (Fig. 3C). In particular, expression cluster V, which is highly enriched with immune genes, was affected by both the Cactus silencing and dengue infection treatments. Differential gene expression in Cactus-silenced and dengue-infected mosquitoes showed a strong correlation with regard to both the direction and magnitude of the regulation of this expression cluster (Fig. 3C, Cluster V).

Further dissection of the expression cluster V defined three main groups: Toll pathway-, JAK-STAT pathway-, and signal modulation- related genes. The signal modulation cascade genes included four C-type lectins (CTLs) and six serine protease inhibitors (SRPNs). A plausible hypothesis is that both the Toll and JAK-STAT pathways may be regulated at least in part by the same signal modulation cascade that includes serine proteases and serpins. Consistent to this hypothesis, evidences suggest that the JAK-STAT pathway could be indirectly activated by the Toll cascade in *D. melanogaster* [30]. Interestingly, genes in this cluster showed similar regulation for the midgut and carcass and for Cactus-silenced mosquitoes, although the magnitude of the regulation was smaller in the midgut, further supporting the notion of a similar type of antiviral defense in the gut and carcass tissues. Expression cluster III was characterized by a repression of seven oxidative defense enzyme genes in both Cactus-silenced and dengue-infected mosquitoes (Fig3C, Cluster III). The genes that showed different profiles for Cactus silencing and dengue infection are listed in the remaining clusters (Fig 3C, Cluster I, II and IV). Several putative apoptotic genes, such as caspases, were also regulated by dengue infection. Similar results have also been observed in *D. melanogaster* in response to *Drosophila* C virus infection [20], suggesting a potential connection between virus infection and apoptosis.

### The Toll pathway is involved in the anti-dengue defense

**Figure 4:**
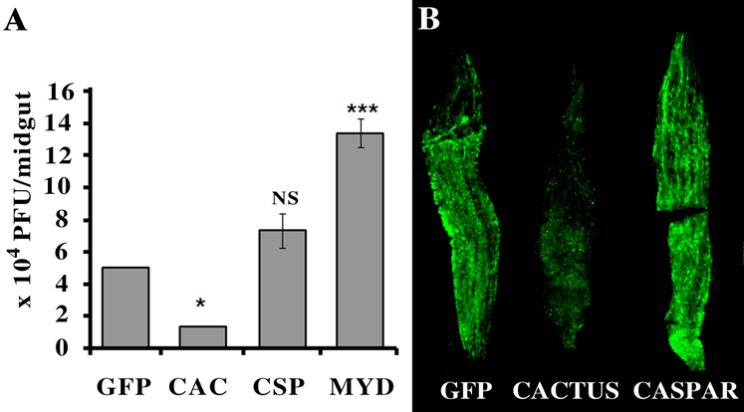
Rel1 regulate anti-dengue activity. Dengue virus loads in the midguts of Cactus, Caspar and MyD88 depleted mosquitoes, and GFP dsRNA treated control mosquitoes. A. Virus titers were measured by plaque assay in C6/36 cell. *, P<0.05, ***, P<0.001, in Student's t-test comparing to GFP control. B. Virus load is assayed through indirect immunofluorescence assay (IFA) in infected midguts. Error bar represents standard error.

The prominent activation of the Toll pathway (Rel1)-regulated genes in response to dengue infection strongly suggested that this pathway is involved in the mosquito's anti-dengue defense. To investigate this hypothesis, we tested the effect of both Cactus and Caspar gene silencing on virus infection in the midgut and carcass at 7 days after an infectious blood meal. This cactus gene silencing reduced the extent of dengue infection in the midgut by 4.0-fold (P<0.05), while Caspar gene silencing had no effect on viral infection when compared to the GFP dsRNA control (Fig. 4). The lower viral loads in the midguts of mosquitoes treated with Cactus dsRNA were also confirmed by IFA assay (Fig. 4). To provide further evidence for Toll pathway implication in controlling dengue virus infection, we assessed whether loss of Toll pathway activation will lead to an increase in virus load. The Toll pathway was inactivated by silencing the MYD88 factor prior to dengue virus titer determination [33]. This resulted in an increase of the virus load by 2.7 times compared to the GFP dsRNA control (P<0.001). Infection levels in the carcass tissue were generally very low for all treatment groups, and there were no significant differences between groups. These results point to a significant role for the Toll pathway in the anti-dengue defense in the midgut tissue and they are similar to those reported for *D. melanogaster*, in which the Toll pathway, but not the Imd pathway, has been shown to be involved in limiting X-virus infection. Together with the gene expression data discussed above, our results suggest that the infection of mosquitoes with dengue virus induces the Toll pathway, which then exerts an anti-dengue effect.

### The mosquito's endogenous bacterial flora influences dengue infection

**Figure 5:**
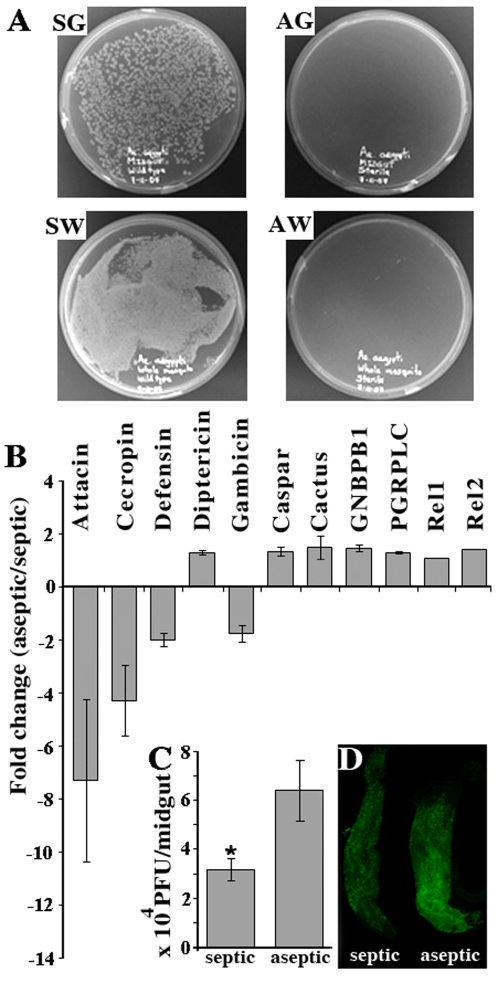
Elimination of the mosquito's endogenous bacteria reduces basal levels of immune gene expression and increases the susceptibility to dengue virus infection. A. LB agar plates at 20 hours after plating homogenized and diluted septic gut (SG) and whole mosquito (SW) from non-treated mosquitoes, and aseptic gut (AG) and whole mosquito (AW) from antibiotic treated mosquitoes B. fold change in the expression of selected immune genes in aseptic mosquitoes compared to septic mosquitoes; C. virus infection levels in aseptic and septic mosquitoes were measured and compared by plaque assay in C6/36 cells * P<0.05 in Student's t-test. D. Dengue virus distribution and loads in septic and aseptic mosquito midguts assayed through IFA. Error bar represents standard error. Primary data for the real-time qPCR assays are presented in Table S2.

The mosquito's innate immune system is mainly involved in the defense against microbes, including control of the mosquito's natural bacterial flora [34], which have also been shown to influence the mosquito's susceptibility to pathogens such as malaria [35],[36] and are responsible for a certain level of basal level activation of immune signaling pathways (Dimopoulos lab, unpublished data). In order to assess the potential influence of the endogenous bacterial flora of *Ae. aegypti* on the mosquito's immune gene expression and susceptibility to dengue virus infection, we compared transcription and infection levels between normal septic mosquitoes and mosquitoes from which the bacterial flora had been eliminated through antibiotic treatment (Fig. 5). The virus titer in the midguts of antibiotic-treated mosquitoes was two times higher than that in the non-treated mosquitoes at 7 days PBM (Fig. 5C and 5D).

This antiviral effect could reflect either a direct interaction between the bacteria and the virus in the midgut lumen or an indirect effect involving a bacteria-elicited basal level of immune activation. To investigate whether the endogenous bacteria flora had the capacity to activate genes regulated by the Toll pathway, we compared the expression levels of 11 selected immune genes, including several antimicrobial peptide genes, in septic and antibiotic-treated aseptic mosquitoes. This analysis showed an elevated expression of several immune marker genes, including the Toll pathway-regulated antimicrobial peptide genes defensin, cecropin, attacin, and gambicin, in the septic mosquitoes, suggesting that the endogenous bacterial flora were stimulating immune gene expression (Fig. 5B). In order to determine whether the midgut bacteria could exert a direct effect on the virus in the midgut lumen, we assayed virus viability after a 3 hrs incubation in the lumenal blood meals of septic and antibiotic-treated aseptic mosquitoes. In a parallel assay, we assessed the loss of virus viability after 4 hrs incubation *in vitro* with the enriched bacteria derived from mosquito midguts. These assays did not show any influence of the bacteria on virus viability (Figure S1), suggesting that the effect of the mosquito's endogenous bacterial flora on dengue virus infection was indirect and likely to be mediated by the mosquito's innate immune system.

## Discussion

In order to dissect the *Ae. aegypti* mosquito responses to dengue virus infection, we have determined the changes in the midgut and carcass transcriptomes that occur upon systemic virus infection at 10 days after an infectious blood meal. These responses involved a variety of functional gene classes, indicating a significant impact of virus infection on mosquito physiology. The broader and stronger response in the carcass is probably related to the late stage of infection when the virus has reached its peak level in the tissues of this compartment; at the same time, infection of the midgut is declining. This analysis revealed a strong bias in the transcriptional response toward genes that have been linked to the Toll immune pathway, and to a lesser extent the JAK-STAT immune pathway, whereas genes putatively linked to the Imd immune pathway seemed to be largely unaffected. A more detailed comparison of the dengue virus infection-responsive transcriptome and the putative Rel1- and Rel2-regulated transcriptomes further corroborated this finding.

The activation of the Toll immune signaling pathway by dengue infection is strongly supported by the up-regulation of Rel1 and several of its upstream putative PRRs and its downstream antimicrobial peptides (Fig. 2). Up-regulation of the *Ae. aegypti* Rel1 (DR081921) has also been observed in response to Sindbis virus infection in a previous study [24], suggesting that these viruses may induce similar responses. In *D. melanogaster*, infection with either the *Drosophila*×virus or *E. coli* has been shown to induce the same antimicrobial peptide genes, suggesting that these two diverse classes of pathogen can activate the same immune response pathway [21]. Infection of *D. melanogaster* with the *Drosophila* C virus also resulted in the up-regulation of several putative Toll pathway-related genes, such as Spätzle, Dorsal, the immune induced molecule 2, CG16836, PGRP-SA, a GNBP-like molecule encoded by CG12780, Drosomycin, and nine other putative antimicrobial peptides [20]. Both PGRP-SA and GNBPs have been linked to the Toll immune signaling pathway; a *D. melanogaster* GNBP functions as a co-receptor for Gram-positive bacteria and is involved in Toll immune pathway activation. It will be interesting to elucidate the functional significance of the observed GNBP induction in response to dengue infection in *Ae. aegypti*. For instance, the GNBPs might function as receptors that interact with the virus or cellular debris released during virus particle release, either activating an immune response or directly neutralizing the virus particles.

Activation of Toll pathway-controlled Rel1 transcription factors through the RNAi-mediated depletion of Cactus resulted in suppression of the viral infection in the midgut, while activation of the Imd pathway-controlled Rel2, mediated through Caspar depletion, had no effect on infection. Although Rel1 over-expressing transgenic mosquitoes have been developed, we chose to use this RNAi-based approach to activate these factors in order to achieve systemic pathway activation and enrichment of immune factors prior to infection. The Rel1 transgenes are under the control of a blood meal-induced fatbody-specific promoter that only activates transcription at around 12 to 20 h after a blood meal in a very specific tissue compartment, which may not be the main destination for the dengue virus (A. Raikhel, personal communication) [31].

The lack of an apparent link between the Imd pathway and the anti-dengue defense at 7 to 10 days after an infectious blood meal does not, however, exclude the possibility that this pathway is involved in the anti-viral response at some other stage of infection or in a specific tissue or cell type. However, our results are consistent with the previous report that the Toll pathway, but not the Imd pathway, in *D. melanogaster* is involved in the defense against the *Drosophila* X virus [21]. The anti-dengue effect of the Toll pathway is likely to be stronger than the four-fold change that we obtained through Cactus gene silencing, given that RNAi-mediated depletion of proteins is known to be incomplete and transient in many cases.

Our gene expression data also pointed to an activation of the less characterized JAK-STAT pathway, which has been shown to be activated in response to *Drosophila* C virus infection in *D. melanogaster*, where it is involved in limiting viral infection but, while required, is not sufficient to mount a potent antiviral response [20]. These findings may suggest that an effective anti-viral response in insects requires the activation of more than one complementary defense system.

Two types of anti-viral defense have been proposed to operate in *D. melanogaster*: an inducible response that involves the activation of immune signaling pathways and an intrinsic defense system based on RNAi [23]. Our analysis of dengue infection-responsive gene expression did not identify any RNAi-related genes, consistent with the previous findings regarding *D. melanogaster* responses to *Drosophila* C virus infection [20]. Components of the RNAi defense systems are likely to be constitutively expressed and do therefore not require further transcriptional induction upon viral challenge. Alternatively, the virus may actively suppress transcription of such transcripts as a defense modulating mechanism.

A major role of immune signaling pathways in insects is to protect the organism against the continuous exposure to opportunistic microbes as well as controlling the natural microbiota, such as the gut flora. Several studies have shown the importance of the host microbiota in inducing a basal level of immune activity that enhances the insect's resistance to pathogens. For instance, antibiotic treatment of *An. gambiae* mosquitoes significantly reduces the microbial midgut flora, resulting in a decreased expression of immune genes and an increased susceptibility to *Plasmodium* infection [35],[36]. Moreover, *Plasmodium* development has recently been shown to be significantly influenced by the mosquito's basal level immunity rather than an infection-stimulated induction of immune genes [13]. In addition, the *An. gambiae* genes implicated in the defense against *Plasmodium* have also been shown to play a role in its anti-bacterial defense [34]. In *Culex. bitaeniorhynchus*, tetracycline treatment resulted in an increased susceptibility to the Japanese encephalitis virus [37]. Similarly, we have observed that aseptic *Ae. aegypti* were less resistant to dengue virus, and they expressed lower levels of certain immune genes that are also controlled by the Toll pathway.

These findings suggest that it is plausible to hypothesize that the mosquito's endogenous microbiota and natural microbial exposure stimulate a certain level of immune gene expression through the activation of the Toll pathway which, in turn, mediates antiviral activity. Alternatively, the microbiota could affect viral infection in a more direct way by altering the biochemical environment of the midgut or by directly interacting with the virus particles. However, the latter is not supported by our preliminary data on virus-bacteria interaction, while a possibility still exist that the bacteria hinder virus interaction with the midgut epithelium (Figure S1). Regardless of their biological basis, however, our results suggest that the microbial exposure in nature is likely to play an important role in modulating the mosquito's anti-viral defense system and its level of resistance to infection. The ability of the Toll immune pathway to suppress dengue infection suggests that it regulates one or several anti-viral effector molecules that remain to be discovered. The level of functional overlap that was observed between anti-bacterial and anti-*Plasmodium* effector genes in *A. gambiae* is unlikely to occur between the anti-microbial and anti-dengue defense systems because of the drastically difference in surface molecules and life style between virus particles and bacteria or parasites; nevertheless, the defenses appear to be regulated by the same immune signaling pathways. Finally, it is unlikely that the presence of low levels of antibiotics in the mosquito hemolymph contributed directly to changes in gene expression or increased virus infection. Antibiotic treatment has for instance no effect on virus propagation in the C6/36 cell line, and experiments in sterile *A. gambiae* mosquito cell lines have not shown any direct effects of antibiotic treatment on immune gene expression (Dimopoulos lab, unpublished data).

In summary, the results presented here show that the Toll pathway is involved in controlling dengue virus infection in *Ae. aegypti*. Dengue infection can activate this pathway, which in turn induces a mechanism that suppresses the virus infection. Consistent with this observation, a low basal immunity in aseptic mosquitoes is correlated with a high virus infection level. Our results provide support for future experiments to dissect the biological network involved in the defense against dengue virus infection in *Ae. aegypti*. For example, it will be interesting to study the potential links between the Toll, JAK-STAT and RNAi pathways and their relevant contribution to the resistance of dengue infection in mosquitoes. In addition, how the virus is recognized to activate the Toll pathway and what are the downstream anti-viral effector molecules are important questions to be answered. In the present study, we have utilized a specific New Guinea genotype of the DENV-2 serotype that is known to be highly virulent. It will be interesting to assess the potential similarities and differences in the response of the mosquito to different dengue virus serotypes and genotypes that are known to differ in their virulence and tropism. Conversely, a comparison of mosquito strains with different susceptibilities to a particular virus genotype may also further our understanding of this complex vector-pathogen system.

In summary, we demonstrate a significant role of the Toll pathway in regulating resistance to dengue virus in *Ae. aegypti*, as indicated by an infection-responsive regulation and functional assessment of several Toll pathway-associated genes. We have also shown that the mosquito's natural microbiota play a role in modulating the dengue virus infection, possibly through basal-level stimulation of the mosquito's antiviral immune system.

## Materials and Methods

### Mosquito rearing and cell culture maintenance

*Ae. aegypti* mosquitoes of the Rockefeller/UGAL strain were maintained on sugar solution at 27°C and 95% humidity with a 12-hr light/dark cycle according to standard rearing procedures. The *Ae. albopictus* cell line C6/36 was grown in minimal essential medium (MEM) with 10% heat inactivated FBS, 1% L-glutamine, and 1% non-essential amino acids at 32°C with 5% CO_2_.

### DENV-2 infections

The New Guinea C strain of DENV-2 was propagated in C6/36 cells according to standard conditions [38]: In brief, 0.5 ml aliquots of virus stock were used to infect 75-cm^2^ flasks of C6/36 cells at 80% confluency with a multiplicity of infection (MOI) of 3.5 virus particles/cell. Infected cells were incubated for 5–7 days. Cells were harvested with a cell scraper and lysed to release the virus particles by repeated freezing and thawing in dry CO_2_ and a 37°C water bath. The virus suspension was mixed 1∶1 with commercial human blood. A flask with uninfected C6/36 cells were maintained under similar conditions and used to create the noninfectious blood meal that served as our control. The blood meal was maintained at 37°C for 30 min prior to feeding 3- to 4-day-old mosquitoes (http://www.jove.com/index/Details.stp?ID=220). Primary PFU data are presented in Table S4.

### Mosquito dissections

For microarray assays, mosquitoes at 10 days after blood meal were dissected to collect the midguts and carcass in RNALater, with 10 to 15 individuals in a single replicate. Three or four replicate biological assays were performed. Total RNA was extracted using the RNeasy kit (QIAGEN), and RNA concentrations were measured using Nanodrop; for virus titer measurement, mosquitoes at 7 days after blood meal were briefly washed in 70% ethanol, then rinsed in sterile distilled water. The midgut and carcass were dissected in sterile PBS and transferred separately to microcentrifuge tubes containing 150 µl of MEM, then homogenized with a Kontes pellet pestle motor in a sterile environment.

### Microarray assays

Transcription assays were conducted and analyzed as reported previously with a full genome Agilent-based microarray platform [28],[34] In brief, 2–3 µg total RNA was used for probe synthesis of cy3- and cy5-labeled dCTP. Hybridizations were conducted with an Agilent Technologies In Situ Hybridization kit at 60°C according to the manufacturer's instructions. Hybridization intensities were determined with an Axon GenePix 4200AL scanner, and images were analyzed with Gene Pix software. The expression data were processed and analyzed as described previously [28]. In brief, the background-subtracted median fluorescent values were normalized according to a LOWESS normalization method, and Cy5/Cy3 ratios from replicate assays were subjected to *t*-tests at a significance level of p<0.05 using TIGR MIDAS and MeV software. Expression data from all replicate assays were averaged with the GEPAS microarray preprocessing software prior to logarithm (base 2) transformation. Self-self hybridizations have been used to determine the cut-off value for the significance of gene regulation on these type of microarrays to 0.8 in log2 scale, which corresponds to 1.74- fold regulation. For genes with P<0.05, the average ratio was used as the final fold change; for genes with P>0.05, the inconsistent replicates (with distance to the median of replicate ratios large than 0.8) were removed, and only the value from a gene with at least two replicates were further averaged. Toll and Imd signaling pathways were built on the basis of a recent bioinformatics prediction [17] with GeneMAPP2 software [39]. The latter was also used for the generation of the expression datasets. The gene database was created with the *Ae. aegypti* gene ontology by the GeneMapp development team. Three independent biological replicate assays were performed. Numeric microarray gene expression data are presented in Tables S1 and S2.

### Real-time qPCR assays

Real-time qPCR assays were conducted as previous described [37]. Briefly, RNA samples were treated with Turbo DNase (Ambion, Austin, Texas, United States) and reverse-transcribed using Superscript III (Invitrogen, Carlsbad, California, United States) with random hexamers. Real-time quantification was performed using the QuantiTect SYBR Green PCR Kit (Qiagen) and ABI Detection System ABI Prism 7000 (Applied Biosystems, Foster City, California, United States). Three independent biological replicates were conducted and all PCR reactions were performed in triplicate. The ribosomal protein S7 gene was used for normalization of cDNA templates. Primer sequences are listed in Table S5. Numeric data for the real-time qPCR assays are presented in Table S3.

### Gene-silencing assays

RNA interference (RNAi)-based gene-silencing assays were conducted according to standard methodology [34]: Approximately 69 ηl dsRNAs (3 µg/µl) in water was injected into the thorax of cold-anesthetized 4-day-old female mosquitoes using a nano-injector as previously described (http://www.jove.com/index/Details.stp?ID=230). Three to four days after injection and validation of gene-specific silencing, mosquitoes were fed on a DENV-2-supplemented blood meal. Dissection of mosquito midguts, thoraxes, and heads were done on the seventh day PBM. Each tissue was homogenized separately in the same medium as used for C6/36 cells (MEM) and used for virus titration. Three independent biological replicate assays were performed for each gene. The following primers were used for the synthesis of Cactus, Caspar and MyD88 dsRNA using the T7 megascript kit (Ambion): Cactus_F: TAATACGACTCACTATAGGG CGAGTCAACAGAACCCGAGCAG, Cactus_R: TAATACGACTCACTATAGGG TGGCCCGTCAGCACCGAAAG, Caspar_F: TAATACGACTCACTATAGGG GGAAGCAGATCGAGCCAAGCAG, Caspar_R: TAATACGACTCACTATAGGG GCATTGAGCCGCCTGGTGTC, MyD88_F: TAATACGACTCACTATAGGGGGCGATTGGTGGTTGTTATT, MyD88_R: TAATACGACTCACTATAGGGTTGAGCGCATTGCTAACATC,

### DENV-2 virus titration

Virus titers in the tissue homogenates were measured as previously reported (http://www.jove.com/index/Details.stp?ID=220): The virus-containing homogenates were serially diluted and inoculated into C6/36 cells in 24-well plates. After incubation for 5 days at 32°C and 5% CO_2_, the plates were assayed for plaque formation by peroxidase immunostaining, using mouse hyperimmune ascitic fluid (MHIAF, specific for DENV-2) and a goat anti-mouse HRP conjugate as the primary and secondary antibody, respectively. Numeric PFU data are presented in Table S3.

### Mosquito antibiotic treatment

After pupation, mosquitoes were transferred to a sterile cage and provided a sterile 10% sucrose solution with 15 mg/ml gentamicin, 10 units penicillin, and 10 µg streptomycin as a sugar source. The removal of microbes was confirmed by colony-forming unit assays prior to blood-feeding and after a surface sterilization that involved vortexing in 70% ethanol and subsequent rinsing in double-distilled sterile H_2_O. Each entire mosquito was then homogenized in 100 µl autoclaved PBS and plated on LB-agar, and the plates were checked for presence of bacterial growth at 48 h post-inoculation.

### Indirect immunofluorescence assays

These assays were performed according to a modification of a previously established method [40]. The midguts from 7-day-old mosquitoes were dissected in 1.0% paraformaldehyde in PBS. After a 1-h incubation in 50 µl of 4.0% paraformaldehyde in a 96-well plate, the midguts were washed three times with 100 µl PBS for 1 min each; 100 µl of 10% goat serum was then added to the antibody dilution buffer (0.1% TritonX-100 and 0.2% BSA in PBS) and incubated overnight. The midguts were then incubated with FITC-conjugated monoclonal antibody 2H2 at 37°C for 1 h. The midguts were washed twice with PBS at room temperature for 1 h and then stained with Evans blue counter-stain (diluted 1: 100), placed onto slides, and covered with Bartel B 1029-45B mounting medium and a coverslip. Preparations were examined under a Nikon fluorescence microscope.

### Accession numbers

The Entrez Gene ID for genes and proteins mentioned in the text are 5565922 (Cactus), 5569526 (REL1A), 5578608 (Caspar), 5569427 (REL2), 5579094 (DEF), 5579377 (CEC), 5578028 (Attacin), 5565542 (Diptericin), 5579192 (GNBPB1), 5564897 (PGRGLC), 5564993 (Gambicin), 5569574 (MyD88), 5579458 (LYSC), 5576410 (Ikkg), 5565422 (GNBPA2), 5580019 (AAEL009645), 5572476 (AAEL009822), 5576330 (AAEL000393), 5576380 (DOME), 5573010 (SPZ5), 5578273 (TOLL1B), 5577966 (TOLL9A), 5576030 (TEP13), 5565197 (TEP15), 5572428 (TEP20). 5563609 (TEP22), 5568254 (FREP), 5577659 (CLIPB13B).

## Supporting Information

Figure S1 A. The bacteria flora in the mosquito lumen does not influence the viability of the dengue virus. Seven days old antibiotic treated aseptic or non-treated septic mosquitoes were fed with the same mixture of DENV-2 and blood. Two hour after the blood meal, midguts were dissected and their content was immediately diluted with 100 ul sterile PBS. Three replicates of five guts each were collected. After a brief homogenization and centrifugation, the supernatants were used to determine the virus titer with the standard plaque assay. B. *In vitro* exposure of dengue virus to midgut bacteria does not affect the virus viability. Incubation of the dengue virus with either sterile PBS, bacteria exposed supernatant or a bacteria suspension did not result in any significant difference in virus viability. Ten midguts from seven days old septic female mosquitoes were dissected and homogenized in 100 ul sterile PBS prior to plating on a LB agar plate for bacterial growth. Bacteria colonies were washed off the plate with PBS and collected into a 1.5 ml tube. After a 10 minutes centrifugation at 1,500 g the bacteria-free supernatant and the bacteria pellet were collected. The bacteria pellet was re-suspended into PBS to get the bacteria solution. Then, equal amount of virus were incubated for 3 hrs at room temperature with the bacteria, the bacteria free supernatant and the sterile PBS prior to titer determination with plaques assay. Three replicates were performed for each treatment.
(0.07 MB JPG)

Table S1 The functional groups of the total 432 genes that were regulated by DENV-2 infection in the mosquito carcass at ten days after an infected blood meal, compared to that of non-infected blood fed control mosquitoes. Functional group abbreviations: IMM, immunity; RED/STE, redox and oxidoreductive stress; CSR, chemosensory reception; DIG, blood and sugar food digestive; PROT, proteolysis; CYT/STR, cytoskeletal and structural; TRP, transport; R/T/T, replication, transcription, and translation; MET, metabolism; DIV, diverse functions; UNK, unknown functions.
(0.38 MB DOC)

Table S2 The functional groups of the total 63 genes that were regulated by DENV-2 infection in the mosquito midgut at ten days after an infected blood meal, compared to that of non-infected blood fed control mosquitoes. Functional group abbreviations: IMM, immunity; RED/STE, redox and oxidoreductive stress; CSR, chemosensory reception; DIG, blood and sugar food digestive; PROT, proteolysis; CYT/STR, cytoskeletal and structural; TRP, transport; R/T/T, replication, transcription, and translation; MET, metabolism; DIV, diverse functions; UNK, unknown functions.
(0.08 MB DOC)

Table S3 Averaged data from three biological replicate real time qPCR assays of the expression of defensin, cecropin, Cactus, and Rel1in Cactus, and Cactus & Rel1 depleted mosquitoes (A) and in Caspar, and Caspar & Rel2 depleted mosquitoes (B). C. Fold change in the expression of selected immune genes in aseptic mosquitoes compared to septic mosquitoes. S.E., standard error.
(0.06 MB DOC)

Table S4 A. Averaged data from three independent biological replicate plaque assays of the virus titer in the midguts of the Cactus, Caspar, MYD88 and GFP dsRNA treated mosquitoes. B. Results from three independent biological replicate plaque assays of the virus titer in the midgut of antibiotic treated aseptic and non-treated septic mosquitoes. S.E., standard error; S, significant; NS, Non-significant.
(0.04 MB DOC)

Table S5 The prime sequences used for the real-time qPCR assays.
(0.04 MB DOC)

Table S6 The expression data of all the genes that are shown in the hierarchical cluster matrix (Fig. 3C).
(0.30 MB DOC)

Text S1 This section refers to other dengue infection responsive genes.
(0.05 MB DOC)
