# Supplementary figures and images for: The Aedes aegypti Toll Pathway Controls Dengue Virus Infection
Source: PLoS Pathog. 2008 Jul 4;4(7):e1000098. doi: 10.1371/journal.ppat.1000098 (PMC2435278; doi:10.1371/journal.ppat.1000098)

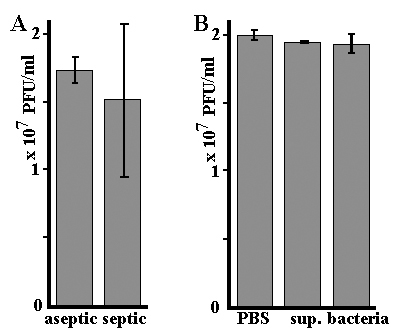

Supplement: Figure S1 — A. The bacteria flora in the mosquito lumen does not influence the viability of the dengue virus. Seven days old antibiotic treated aseptic or non-treated septic mosquitoes were fed with the same mixture of DENV-2 and blood. Two hour after the blood meal, midguts were dissected and their content was immediately diluted with 100 ul sterile PBS. Three replicates of five guts each were collected. After a brief homogenization and centrifugation, the supernatants were used to determine the virus titer with the standard plaque assay. B. In vitro exposure of dengue virus to midgut bacteria does not affect the virus viability. Incubation of the dengue virus with either sterile PBS, bacteria exposed supernatant or a bacteria suspension did not result in any significant difference in virus viability. Ten midguts from seven days old septic female mosquitoes were dissected and homogenized in 100 ul sterile PBS prior to plating on a LB agar plate for bacterial growth. Bacteria colonies were washed off the plate with PBS and collected into a 1.5 ml tube. After a 10 minutes centrifugation at 1,500 g the bacteria-free supernatant and the bacteria pellet were collected. The bacteria pellet was re-suspended into PBS to get the bacteria solution. Then, equal amount of virus were incubated for 3 hrs at room temperature with the bacteria, the bacteria free supernatant and the sterile PBS prior to titer determination with plaques assay. Three replicates were performed for each treatment. (0.07 MB JPG) [file ppat.1000098.s001.jpg]
