# Supplementary material for: The Aedes aegypti Toll Pathway Controls Dengue Virus Infection
Source: PLoS Pathog. 2008 Jul 4;4(7):e1000098. doi: 10.1371/journal.ppat.1000098 (PMC2435278; doi:10.1371/journal.ppat.1000098)
Supplement: Table S1 — The functional groups of the total 432 genes that were regulated by DENV-2 infection in the mosquito carcass at ten days after an infected blood meal, compared to that of non-infected blood fed control mosquitoes. Functional group abbreviations: IMM, immunity; RED/STE, redox and oxidoreductive stress; CSR, chemosensory reception; DIG, blood and sugar food digestive; PROT, proteolysis; CYT/STR, cytoskeletal and structural; TRP, transport; R/T/T, replication, transcription, and translation; MET, metabolism; DIV, diverse functions; UNK, unknown functions. (0.38 MB DOC) [file ppat.1000098.s002.doc]

| **Gene Name** | **Gene ID** | **Functional Group** | **Average log2 value** |
| --- | --- | --- | --- |
| tubulin alpha chain | AAEL006642 | CS | -1.25 |
| myosin heavy chain, nonmuscle or smooth muscle | AAEL001411 | CS | -0.94 |
| growth factor receptor-bound protein | AAEL013786 | CS | -0.81 |
| growth factor receptor-bound protein | AAEL014394 | CS | -0.81 |
| cadherin | AAEL000700 | CS | 1.39 |
| hypothetical protein | AAEL001028 | CS | 1.67 |
| conserved hypothetical protein | AAEL000419 | CS | 2.48 |
| conserved hypothetical protein | AAEL008009 | CSR | 0.85 |
| Odorant receptor 9a, putative | AAEL010410 | CSR | 0.87 |
| bhlhzip transcription factor bigmax | AAEL011202 | DIV | -2.00 |
| conserved hypothetical protein | AAEL003355 | DIV | -1.58 |
| hypothetical protein | AAEL002920 | DIV | -1.46 |
| cdk1 | AAEL012339 | DIV | -1.44 |
| cdk1 | AAEL013329 | DIV | -1.44 |
| hypothetical protein | AAEL009962 | DIV | -1.43 |
| alkaline phosphatase | AAEL000931 | DIV | -1.39 |
| conserved hypothetical protein | AAEL000776 | DIV | -1.19 |
| adenylate cyclase type | AAEL009022 | DIV | -1.13 |
| fructose-bisphosphate aldolase | AAEL005766 | DIV | -1.13 |
| hypothetical protein | AAEL002473 | DIV | -1.10 |
| conserved hypothetical protein | AAEL012551 | DIV | -1.05 |
| cyclin d | AAEL011648 | DIV | -1.05 |
| Thymidylate kinase, putative | AAEL001246 | DIV | -1.05 |
| receptor for activated C kinase, putative | AAEL011892 | DIV | -1.01 |
| amidophosphoribosyltransferase | AAEL003581 | DIV | -0.99 |
| yellow protein precursor, putative | AAEL014001 | DIV | -0.97 |
| conserved hypothetical protein | AAEL012865 | DIV | -0.97 |
| serine hydroxymethyltransferase | AAEL002510 | DIV | -0.97 |
| cell division cycle 20 (cdc20) (fizzy) | AAEL014025 | DIV | -0.97 |
| conserved hypothetical protein | AAEL011250 | DIV | -0.96 |
| hypothetical protein | AAEL010818 | DIV | -0.95 |
| conserved hypothetical protein | AAEL005522 | DIV | -0.95 |
| niemann-pick C1 | AAEL003325 | DIV | -0.95 |
| geminin, putative | AAEL009773 | DIV | -0.95 |
| spingomyelin synthetase | AAEL004710 | DIV | -0.94 |
| conserved hypothetical protein | AAEL005300 | DIV | -0.94 |
| hypothetical protein | AAEL003465 | DIV | -0.94 |
| KEY | AAEL012510 | DIV | -0.91 |
| conserved hypothetical protein | AAEL013749 | DIV | -0.91 |
| hypothetical protein | AAEL012085 | DIV | -0.91 |
| conserved hypothetical protein | AAEL015080 | DIV | -0.90 |
| translocon-associated protein, delta subunit | AAEL013320 | DIV | -0.90 |
| hypothetical protein | AAEL008686 | DIV | -0.90 |
| serine/threonine protein kinase | AAEL000217 | DIV | -0.89 |
| regulator of chromosome condensation | AAEL007799 | DIV | -0.89 |
| conserved hypothetical protein | AAEL013912 | DIV | -0.88 |
| zinc finger protein | AAEL002388 | DIV | -0.87 |
| zinc finger protein | AAEL012224 | DIV | -0.87 |
| hypothetical protein | AAEL010899 | DIV | -0.87 |
| ras-related protein, putative | AAEL010430 | DIV | -0.87 |
| Inhibitor of growth protein, ing1 | AAEL003650 | DIV | -0.86 |
| conserved hypothetical protein | AAEL005631 | DIV | -0.86 |
| conserved hypothetical protein | AAEL011295 | DIV | -0.86 |
| purine biosynthesis protein 6, pur6 | AAEL003606 | DIV | -0.85 |
| conserved hypothetical protein | AAEL010762 | DIV | -0.85 |
| spermatogenesis associated factor | AAEL010585 | DIV | -0.85 |
| hypothetical protein | AAEL009645 | DIV | -0.85 |
| type II collagen, putative | AAEL009812 | DIV | -0.84 |
| conserved hypothetical protein | AAEL004699 | DIV | -0.84 |
| somatostatin receptor | AAEL012356 | DIV | -0.84 |
| phosphatidylserine receptor | AAEL008084 | DIV | -0.83 |
| scaffold attachment factor b | AAEL001352 | DIV | -0.83 |
| conserved hypothetical protein | AAEL007848 | DIV | -0.83 |
| conserved hypothetical protein | AAEL014844 | DIV | -0.83 |
| conserved hypothetical protein | AAEL002495 | DIV | -0.83 |
| conserved hypothetical protein | AAEL011714 | DIV | -0.83 |
| sentrin/sumo-specific protease | AAEL008952 | DIV | -0.82 |
| hypothetical protein | AAEL011141 | DIV | -0.82 |
| conserved hypothetical protein | AAEL010905 | DIV | -0.81 |
| hypothetical protein | AAEL007833 | DIV | 0.80 |
| conserved hypothetical protein | AAEL013797 | DIV | 0.80 |
| electron transfer flavoprotein-ubiquinone oxidoreductase | AAEL007526 | DIV | 0.80 |
| frizzled, putative | AAEL006832 | DIV | 0.80 |
| conserved hypothetical protein | AAEL011069 | DIV | 0.81 |
| conserved hypothetical protein | AAEL006519 | DIV | 0.81 |
| conserved hypothetical protein | AAEL012635 | DIV | 0.81 |
| lethal(2)essential for life protein, l2efl | AAEL010659 | DIV | 0.81 |
| lethal(2)essential for life protein, l2efl | AAEL013343 | DIV | 0.81 |
| WAP four-disulfide core domain protein 2 precursor, putative | AAEL011639 | DIV | 0.82 |
| mical | AAEL005439 | DIV | 0.82 |
| serine/threonine protein kinase, putative | AAEL007688 | DIV | 0.83 |
| hypothetical protein | AAEL000236 | DIV | 0.84 |
| conserved hypothetical protein | AAEL012566 | DIV | 0.84 |
| conserved hypothetical protein | AAEL002896 | DIV | 0.85 |
| tnf receptor associated factor | AAEL006649 | DIV | 0.86 |
| adenosine kinase | AAEL001856 | DIV | 0.86 |
| hypothetical protein | AAEL003549 | DIV | 0.86 |
| secreted modular calcium-binding protein | AAEL012043 | DIV | 0.86 |
| conserved hypothetical protein | AAEL003425 | DIV | 0.86 |
| g-protein-linked acetylcholine receptor gar-2a | AAEL007832 | DIV | 0.87 |
| g-protein-linked acetylcholine receptor gar-2a | AAEL015037 | DIV | 0.87 |
| conserved hypothetical protein | AAEL001420 | DIV | 0.87 |
| ultraviolet-sensitive opsin | AAEL009615 | DIV | 0.87 |
| ecdysone-induced protein 75b | AAEL007397 | DIV | 0.88 |
| conserved hypothetical protein | AAEL000153 | DIV | 0.89 |
| hypothetical protein | AAEL008015 | DIV | 0.89 |
| conserved hypothetical protein | AAEL013552 | DIV | 0.90 |
| ubiquitin-conjugating enzyme h | AAEL013633 | DIV | 0.90 |
| conserved hypothetical protein | AAEL005083 | DIV | 0.90 |
| circadian locomoter output cycles kaput protein (dclock) (dpas1) | AAEL012562 | DIV | 0.91 |
| conserved hypothetical protein | AAEL000580 | DIV | 0.91 |
| synaptojanin | AAEL011417 | DIV | 0.91 |
| forkhead box protein (AaegFOXM2) | AAEL000041 | DIV | 0.91 |
| conserved hypothetical protein | AAEL000945 | DIV | 0.91 |
| conserved hypothetical protein | AAEL002355 | DIV | 0.96 |
| conserved hypothetical protein | AAEL009230 | DIV | 0.97 |
| semaphorin | AAEL002653 | DIV | 0.97 |
| numb-associated kinase | AAEL009305 | DIV | 0.97 |
| conserved hypothetical protein | AAEL005982 | DIV | 0.98 |
| hypothetical protein | AAEL003574 | DIV | 0.98 |
| hypothetical protein | AAEL013040 | DIV | 1.00 |
| arginine or creatine kinase | AAEL000959 | DIV | 1.00 |
| hypothetical protein | AAEL002400 | DIV | 1.01 |
| conserved hypothetical protein | AAEL009382 | DIV | 1.02 |
| conserved hypothetical protein | AAEL008320 | DIV | 1.02 |
| mediator complex, subunit, putative | AAEL005934 | DIV | 1.02 |
| multicopper oxidase | AAEL001667 | DIV | 1.03 |
| hypothetical protein | AAEL007073 | DIV | 1.03 |
| werner syndrome helicase | AAEL003152 | DIV | 1.04 |
| conserved hypothetical protein | AAEL015522 | DIV | 1.05 |
| sap18 | AAEL014368 | DIV | 1.07 |
| molybdopterin biosynthesis moeb protein | AAEL004607 | DIV | 1.08 |
| malic enzyme | AAEL001073 | DIV | 1.09 |
| conserved hypothetical protein | AAEL006087 | DIV | 1.09 |
| conserved hypothetical protein | AAEL006925 | DIV | 1.10 |
| conserved hypothetical protein | AAEL008961 | DIV | 1.11 |
| runt | AAEL006167 | DIV | 1.13 |
| conserved hypothetical protein | AAEL015285 | DIV | 1.14 |
| hypothetical protein | AAEL009087 | DIV | 1.16 |
| modifier of mdg4 | AAEL010576 | DIV | 1.18 |
| conserved hypothetical protein | AAEL013747 | DIV | 1.18 |
| conserved hypothetical protein | AAEL011995 | DIV | 1.18 |
| conserved hypothetical protein | AAEL000529 | DIV | 1.18 |
| conserved hypothetical protein | AAEL004503 | DIV | 1.18 |
| conserved hypothetical protein | AAEL002064 | DIV | 1.21 |
| conserved hypothetical protein | AAEL009589 | DIV | 1.21 |
| cysteine-rich venom protein, putative | AAEL000356 | DIV | 1.21 |
| hypothetical protein | AAEL000503 | DIV | 1.21 |
| somatostatin receptor | AAEL012920 | DIV | 1.22 |
| conserved hypothetical protein | AAEL014002 | DIV | 1.25 |
| expressed protein (HR4) | AAEL005850 | DIV | 1.25 |
| forkhead box protein (AaegFOXN2) | AAEL009900 | DIV | 1.26 |
| conserved hypothetical protein | AAEL000102 | DIV | 1.26 |
| paired box protein, putative | AAEL011647 | DIV | 1.26 |
| Dissatisfaction (Dsf) | AAEL005381 | DIV | 1.28 |
| serine/threonine protein kinase | AAEL009360 | DIV | 1.30 |
| hypothetical protein | AAEL002545 | DIV | 1.34 |
| conserved hypothetical protein | AAEL012105 | DIV | 1.38 |
| hypothetical protein | AAEL007053 | DIV | 1.38 |
| metabotropic glutamate receptor 4, 6, 7, 8 (mglur group 3) | AAEL009822 | DIV | 1.40 |
| hypothetical protein | AAEL013175 | DIV | 1.42 |
| niemann-pick C1 | AAEL009531 | DIV | 1.43 |
| hypothetical protein | AAEL010857 | DIV | 1.43 |
| conserved hypothetical protein | AAEL009841 | DIV | 1.45 |
| conserved hypothetical protein | AAEL010333 | DIV | 1.86 |
| chordin | AAEL005627 | DIV | 1.92 |
| zinc finger protein | AAEL001526 | DIV | 1.95 |
| conserved hypothetical protein | AAEL007408 | DIV | 1.96 |
| rho guanine exchange factor | AAEL013280 | DIV | 2.01 |
| zinc finger protein | AAEL009508 | DIV | 2.04 |
| hypothetical protein | AAEL008839 | DIV | 2.22 |
| serine/threonine-protein kinase vrk | AAEL015216 | DIV | 2.32 |
| conserved hypothetical protein | AAEL007436 | DIV | 2.32 |
| hypothetical protein | AAEL014392 | DIV | 2.44 |
| zinc finger protein | AAEL004458 | DIV | 2.51 |
| TEP | AAEL000087 | IMM | 1.82 |
| conserved hypothetical protein | AAEL000255 | IMM | 0.83 |
| epithelial membrane protein | AAEL000256 | IMM | 1.04 |
| superoxide dismutase | AAEL000274 | IMM | -0.91 |
| suppressors of cytokine signalling | AAEL000393 | IMM | 0.91 |
| BGBP | AAEL000652 | IMM | 0.80 |
| CACTUS | AAEL000709 | IMM | -0.84 |
| hexamerin 2 beta | AAEL000765 | IMM | -0.84 |
| TEP | AAEL001794 | IMM | 0.90 |
| serine protease | AAEL002585 | IMM | 0.83 |
| serine protease | AAEL002595 | IMM | 0.94 |
| serine protease | AAEL002610 | IMM | 1.07 |
| serine protease | AAEL002629 | IMM | 1.12 |
| SRPN | AAEL002730 | IMM | 1.43 |
| conserved hypothetical protein | AAEL003119 | IMM | 0.85 |
| CLIP | AAEL003253 | IMM | 1.04 |
| CASP | AAEL003439 | IMM | 0.80 |
| conserved hypothetical protein | AAEL003832 | IMM | -1.81 |
| conserved hypothetical protein | AAEL003849 | IMM | 0.82 |
| TPx | AAEL004112 | IMM | -1.43 |
| HPx | AAEL004386 | IMM | -1.11 |
| HPx | AAEL004388 | IMM | -1.68 |
| HPx | AAEL004390 | IMM | -1.03 |
| CLIP | AAEL005064 | IMM | -0.85 |
| dopachrome-conversion enzyme (DCE) isoenzyme, putative | AAEL005325 | IMM | -1.05 |
| conserved hypothetical protein | AAEL005443 | IMM | 1.26 |
| serine protease inhibitor, serpin | AAEL005673 | IMM | 0.88 |
| yellow protein precursor | AAEL005738 | IMM | 0.85 |
| programmed cell death | AAEL005832 | IMM | -1.06 |
| SOD-Cu-Zn | AAEL006271 | IMM | -0.84 |
| chymotrypsin, putative | AAEL006383 | IMM | 0.85 |
| clip-domain serine protease, putative | AAEL006576 | IMM | 0.91 |
| FREP | AAEL006702 | IMM | 1.14 |
| gram-negative bacteria binding protein | AAEL007064 | IMM | 0.89 |
| embryonic polarity dorsal | AAEL007696 | IMM | 0.92 |
| SRPN | AAEL008364 | IMM | -0.95 |
| BGBP | AAEL009178 | IMM | 0.92 |
| SOD-Cu-Zn | AAEL009436 | IMM | -0.95 |
| conserved hypothetical protein | AAEL009861 | IMM | 1.11 |
| conserved hypothetical protein | AAEL010973 | IMM | 1.37 |
| CTL | AAEL011455 | IMM | 1.09 |
| SOD-Cu-Zn | AAEL011498 | IMM | -0.90 |
| PGRP | AAEL011608 | IMM | 1.96 |
| galactose-specific C-type lectin, putative | AAEL011619 | IMM | 0.99 |
| hypothetical protein | AAEL011699 | IMM | 0.89 |
| CASP | AAEL012143 | IMM | -0.85 |
| TEP | AAEL012267 | IMM | 1.32 |
| DOME | AAEL012471 | IMM | 1.08 |
| conserved hypothetical protein | AAEL012958 | IMM | 2.49 |
| leucine-rich transmembrane protein | AAEL013441 | IMM | 1.19 |
| hexamerin 2 beta | AAEL013757 | IMM | 1.33 |
| SRPN | AAEL013936 | IMM | 1.35 |
| serine protease inhibitor, serpin | AAEL014078 | IMM | 0.88 |
| SRPN | AAEL014079 | IMM | 0.91 |
| aromatic amino acid decarboxylase | AAEL014238 | IMM | -0.85 |
| galactose-specific C-type lectin, putative | AAEL014390 | IMM | 0.99 |
| TPx | AAEL014548 | IMM | -0.89 |
| TEP | AAEL014755 | IMM | 1.19 |
| peptidoglycan recognition protein-1, putative | AAEL014989 | IMM | 2.11 |
| slit protein | AAEL015322 | IMM | 1.00 |
| LYS | AAEL015404 | IMM | 1.01 |
| antibacterial peptide, putative | AAEL015515 | IMM | 1.05 |
| WASP | Aaeg:N12802 | IMM | 0.90 |
| MBC | Aaeg:N1470 | IMM | 2.41 |
| DDC | Aaeg:N16753 | IMM | -0.99 |
| DCE | Aaeg:N18089 | IMM | -0.85 |
| TEP | Aaeg:N18111 | IMM | 1.24 |
| DCE | Aaeg:N18387 | IMM | -0.91 |
| LRP1 | Aaeg:N19019 | IMM | 1.07 |
| TEP | Aaeg:N19306 | IMM | 0.89 |
| NINAA | Aaeg:N27468 | IMM | 0.87 |
| DSCAM | Aaeg:N31373 | IMM | 1.14 |
| DDC | Aaeg:N32137 | IMM | -0.85 |
| SOCS | Aaeg:N33231 | IMM | 0.81 |
| SOD-Cu-Zn | Aaeg:N34696 | IMM | -0.89 |
| SOD-Cu-Zn | Aaeg:N35244 | IMM | -0.93 |
| DDC | Aaeg:N36360 | IMM | 1.28 |
| SCAR | Aaeg:N38202 | IMM | -1.02 |
| RANK1 | Aaeg:N38609 | IMM | 1.19 |
| FKH | Aaeg:N40610 | IMM | 2.06 |
| DCE | Aaeg:N44512 | IMM | -1.02 |
| DCE | Aaeg:N48367 | IMM | -0.97 |
| SOCS | Aaeg:N49029 | IMM | 1.26 |
| HSC70-3 | Aaeg:N51900 | IMM | -1.00 |
| SOCS | Aaeg:N52152 | IMM | 1.15 |
| DSCAM | Aaeg:N6593 | IMM | 1.05 |
| 4-nitrophenylphosphatase | AAEL007097 | MET | -1.53 |
| deoxyuridine 5'-triphosphate nucleotidohydrolase | AAEL007323 | MET | -1.47 |
| glycerol kinase | AAEL006239 | MET | -1.45 |
| triosephosphate isomerase | AAEL002542 | MET | -1.39 |
| 3-hydroxyisobutyrate dehydrogenase | AAEL010208 | MET | -1.14 |
| phosphoenolpyruvate carboxykinase | AAEL000006 | MET | -1.11 |
| 3-hydroxyisobutyrate dehydrogenase, putative | AAEL009245 | MET | -1.09 |
| glycine rich RNA binding protein, putative | AAEL015143 | MET | -1.08 |
| 3-hydroxyisobutyrate dehydrogenase | AAEL006684 | MET | -1.06 |
| 3-hydroxyisobutyrate dehydrogenase | AAEL012580 | MET | -1.03 |
| Bj1 protein, putative | AAEL013819 | MET | -1.00 |
| selenophosphate synthase | AAEL008849 | MET | -0.93 |
| dolichyl-phosphate beta-D-mannosyltransferase, putative | AAEL003084 | MET | -0.90 |
| dolichyl-phosphate beta-D-mannosyltransferase, putative | AAEL014186 | MET | -0.90 |
| methylenetetrahydrofolate dehydrogenase | AAEL010751 | MET | -0.88 |
| glucosamine-6-phosphate isomerase | AAEL013877 | MET | -0.86 |
| malate dehydrogenase | AAEL008166 | MET | -0.84 |
| paraplegin | AAEL009721 | MET | -0.84 |
| goliath E3 ubiquitin ligase | AAEL012337 | MET | -0.83 |
| lumbrokinase-3(1) precursor, putative | AAEL007593 | MET | -0.82 |
| methionine aminopeptidase | AAEL003769 | MET | -0.81 |
| pre-mrna processing factor | AAEL008416 | MET | 0.80 |
| hydroxymethylglutaryl-coa synthase | AAEL005201 | MET | 1.27 |
| prolylcarboxypeptidase, putative | AAEL012664 | MET | 1.74 |
| host cell factor C1 | AAEL008905 | MET | 1.98 |
| conserved hypothetical protein | AAEL001112 | PROT | 1.45 |
| matrix metalloproteinase | AAEL002655 | PROT | 1.44 |
| hypothetical protein | AAEL006323 | PROT | 1.11 |
| cell cycle checkpoint protein rad17 | AAEL007649 | PROT | 1.06 |
| small calcium-binding mitochondrial carrier, putative | AAEL004589 | RED/STE | -1.53 |
| hypothetical protein | AAEL004400 | RED/STE | -1.24 |
| heat shock protein | AAEL011704 | RED/STE | -1.23 |
| heat shock protein | AAEL014843 | RED/STE | -1.04 |
| heat shock protein | AAEL011708 | RED/STE | -0.88 |
| heat shock protein, putative | AAEL001052 | RED/STE | -0.84 |
| mitochondrial solute carrier | AAEL006362 | RED/STE | -0.84 |
| mitochondrial import inner membrane translocase subunit tim17 | AAEL010002 | RED/STE | -0.83 |
| mitochondrial import inner membrane translocase subunit tim17 | AAEL015575 | RED/STE | -0.83 |
| heat shock protein | AAEL014845 | RED/STE | -0.81 |
| mitochondrial ribosomal protein, S11, putative | AAEL005413 | RED/STE | 0.84 |
| conserved hypothetical protein | AAEL009964 | RED/STE | 0.86 |
| NADH dehydrogenase, putative | AAEL010673 | RED/STE | 0.89 |
| mitochondrial ribosomal protein, S18C, putative | AAEL001615 | RED/STE | 0.89 |
| heat shock factor binding protein, putative | AAEL003215 | RED/STE | 1.19 |
| histone h2a | AAEL012499 | R/T/T | -1.89 |
| histone H4 | AAEL003823 | R/T/T | -1.82 |
| histone H4 | AAEL003833 | R/T/T | -1.82 |
| histone H4 | AAEL003838 | R/T/T | -1.82 |
| histone H4 | AAEL003846 | R/T/T | -1.82 |
| histone H4 | AAEL003863 | R/T/T | -1.82 |
| histone H4 | AAEL003866 | R/T/T | -1.82 |
| histone H3 | AAEL003827 | R/T/T | -1.53 |
| histone H3 | AAEL003828 | R/T/T | -1.53 |
| histone H3 | AAEL003836 | R/T/T | -1.53 |
| histone H3 | AAEL003850 | R/T/T | -1.53 |
| histone H3 | AAEL003852 | R/T/T | -1.53 |
| histone H3 | AAEL003856 | R/T/T | -1.53 |
| dead box atp-dependent rna helicase | AAEL008500 | R/T/T | -1.50 |
| Histone H2A, putative | AAEL015390 | R/T/T | -1.46 |
| histone h2a | AAEL003862 | R/T/T | -1.34 |
| histone H4 | AAEL003673 | R/T/T | -1.32 |
| histone H3 | AAEL011424 | R/T/T | -1.30 |
| histone h2a | AAEL007609 | R/T/T | -1.28 |
| rna and export factor binding protein | AAEL005114 | R/T/T | -1.25 |
| rna and export factor binding protein | AAEL015263 | R/T/T | -1.25 |
| arginine/serine-rich splicing factor | AAEL006473 | R/T/T | -1.21 |
| histone H3 | AAEL003685 | R/T/T | -1.07 |
| eukaryotic translation initiation factor 4 gamma | AAEL007928 | R/T/T | -1.04 |
| serine/arginine rich splicing factor | AAEL010340 | R/T/T | -1.02 |
| dead box atp-dependent rna helicase | AAEL010402 | R/T/T | -1.02 |
| DNA-directed RNA polymerase II 19 kDa polypeptide rpb7 | AAEL003401 | R/T/T | -1.01 |
| 20 Kd nuclear cap binding protein | AAEL006135 | R/T/T | -0.96 |
| histone h2a | AAEL003818 | R/T/T | -0.94 |
| histone h2a | AAEL003820 | R/T/T | -0.94 |
| histone h2a | AAEL003826 | R/T/T | -0.94 |
| histone h2a | AAEL003851 | R/T/T | -0.94 |
| dead box atp-dependent rna helicase | AAEL009913 | R/T/T | -0.93 |
| eukaryotic translation initiation factor 3, theta subunit | AAEL007078 | R/T/T | -0.92 |
| eukaryotic translation initiation factor 4 gamma | AAEL007923 | R/T/T | -0.89 |
| alternative splicing type 3 and, putative | AAEL010612 | R/T/T | -0.89 |
| alternative splicing type 3 and, putative | AAEL011687 | R/T/T | -0.89 |
| dna repair protein xp-c / rad4 | AAEL003893 | R/T/T | -0.82 |
| conserved hypothetical protein | AAEL006883 | R/T/T | -0.80 |
| 60S ribosomal protein L7 | AAEL012585 | R/T/T | 0.89 |
| t-box transcription factor tbx20 | AAEL014429 | R/T/T | 0.93 |
| hypothetical protein | AAEL000098 | R/T/T | 0.94 |
| hypothetical protein | AAEL003372 | R/T/T | 1.08 |
| t-box transcription factor tbx6 | AAEL004174 | R/T/T | 1.18 |
| amino acid transporter | AAEL007458 | TRP | -1.40 |
| cis,cis-muconate transport protein MucK, putative | AAEL011470 | TRP | -1.26 |
| mfs transporter | AAEL013146 | TRP | -1.13 |
| amino acids transporter | AAEL002525 | TRP | -0.98 |
| folate carrier protein | AAEL006879 | TRP | -0.97 |
| mfs transporter | AAEL012183 | TRP | -0.95 |
| diacylglycerol o-acyltransferase | AAEL008878 | TRP | -0.95 |
| zinc transporter | AAEL001968 | TRP | -0.88 |
| cationic amino acid transporter | AAEL009362 | TRP | -0.86 |
| abc transporter | AAEL008138 | TRP | -0.84 |
| nucleoporin | AAEL005635 | TRP | -0.83 |
| ion channel nompc | AAEL011679 | TRP | 0.85 |
| cyclophilin-r | AAEL009421 | TRP | 0.85 |
| copper-transporting atpase 1, 2 (copper pump 1, 2) | AAEL003433 | TRP | 0.92 |
| neurotransmitter gated ion channel | AAEL006526 | TRP | 0.95 |
| Sialin, Sodium/sialic acid cotransporter, putative | AAEL004268 | TRP | 1.21 |
| tricarboxylate transport protein | AAEL005991 | TRP | 1.24 |
| organic cation transporter | AAEL009206 | TRP | 1.68 |
| synaptotagmin-4, | AAEL002756 | TRP | 1.76 |
| clathrin coat assembly protein | AAEL001405 | TRP | 2.38 |
| hypothetical protein | AAEL000160 | UNK | 0.99 |
| hypothetical protein | AAEL000675 | UNK | 0.86 |
| hypothetical protein | AAEL000727 | UNK | 0.82 |
| conserved hypothetical protein | AAEL000896 | UNK | 2.06 |
| hypothetical protein | AAEL000969 | UNK | 1.11 |
| SPZ | AAEL001929 | UNK | 1.61 |
| conserved hypothetical protein | AAEL002095 | UNK | 2.15 |
| conserved hypothetical protein | AAEL002803 | UNK | 1.00 |
| hypothetical protein | AAEL002975 | UNK | 1.64 |
| conserved hypothetical protein | AAEL002979 | UNK | 2.40 |
| conserved hypothetical protein | AAEL003089 | UNK | 1.09 |
| conserved hypothetical protein | AAEL003131 | UNK | 1.10 |
| hypothetical protein | AAEL003316 | UNK | 0.94 |
| conserved hypothetical protein | AAEL003430 | UNK | -0.95 |
| hypothetical protein | AAEL004498 | UNK | 0.89 |
| conserved hypothetical protein | AAEL004522 | UNK | 0.85 |
| hypothetical protein | AAEL004604 | UNK | 0.84 |
| conserved hypothetical protein | AAEL004625 | UNK | 1.05 |
| conserved hypothetical protein | AAEL004734 | UNK | 2.75 |
| hypothetical protein | AAEL004754 | UNK | 0.93 |
| conserved hypothetical protein | AAEL004976 | UNK | -1.57 |
| conserved hypothetical protein | AAEL005121 | UNK | 1.77 |
| hypothetical protein | AAEL005192 | UNK | -2.22 |
| conserved hypothetical protein | AAEL005389 | UNK | 0.80 |
| conserved hypothetical protein | AAEL006001 | UNK | 1.12 |
| hypothetical protein | AAEL006072 | UNK | 0.94 |
| hypothetical protein | AAEL006243 | UNK | -1.70 |
| conserved hypothetical protein | AAEL006247 | UNK | -1.07 |
| conserved hypothetical protein | AAEL006502 | UNK | 1.28 |
| hypothetical protein | AAEL006606 | UNK | 2.19 |
| conserved hypothetical protein | AAEL006755 | UNK | 1.15 |
| hypothetical protein | AAEL007744 | UNK | 1.03 |
| hypothetical protein | AAEL007940 | UNK | 2.09 |
| conserved hypothetical protein | AAEL008439 | UNK | -0.86 |
| conserved hypothetical protein | AAEL008492 | UNK | 2.52 |
| conserved hypothetical protein | AAEL008636 | UNK | 0.99 |
| hypothetical protein | AAEL008709 | UNK | -1.29 |
| hypothetical protein | AAEL009040 | UNK | 1.52 |
| hypothetical protein | AAEL009070 | UNK | 1.04 |
| hypothetical protein | AAEL009082 | UNK | 1.18 |
| conserved hypothetical protein | AAEL009247 | UNK | 1.34 |
| hypothetical protein | AAEL009322 | UNK | 0.86 |
| hypothetical protein | AAEL009385 | UNK | 0.87 |
| conserved hypothetical protein | AAEL009473 | UNK | 0.93 |
| conserved hypothetical protein | AAEL009565 | UNK | 1.14 |
| hypothetical protein | AAEL010022 | UNK | -0.82 |
| conserved hypothetical protein | AAEL010113 | UNK | 1.11 |
| hypothetical protein | AAEL010155 | UNK | 1.03 |
| conserved hypothetical protein | AAEL010407 | UNK | -1.12 |
| conserved hypothetical protein | AAEL010898 | UNK | 1.48 |
| hypothetical protein | AAEL011737 | UNK | -0.95 |
| hypothetical protein | AAEL011771 | UNK | 1.00 |
| conserved hypothetical protein | AAEL011826 | UNK | 0.82 |
| conserved hypothetical protein | AAEL011872 | UNK | -1.06 |
| hypothetical protein | AAEL012058 | UNK | 1.25 |
| hypothetical protein | AAEL012504 | UNK | -0.82 |
| conserved hypothetical protein | AAEL012742 | UNK | -0.87 |
| hypothetical protein | AAEL012754 | UNK | 1.18 |
| hypothetical protein | AAEL013024 | UNK | 2.18 |
| conserved hypothetical protein | AAEL013037 | UNK | 1.28 |
| conserved hypothetical protein | AAEL013169 | UNK | 1.22 |
| predicted protein | AAEL013776 | UNK | 0.94 |
| SRPN | AAEL013934 | UNK | 1.34 |
| conserved hypothetical protein | AAEL013977 | UNK | -0.99 |
| hypothetical protein | AAEL014126 | UNK | -2.33 |
| conserved hypothetical protein | AAEL014294 | UNK | -0.82 |
| conserved hypothetical protein | AAEL014585 | UNK | -0.99 |
| hypothetical protein | AAEL014816 | UNK | -0.91 |
| conserved hypothetical protein | AAEL014962 | UNK | 0.87 |
| hypothetical protein | AAEL015118 | UNK | 0.87 |
| hypothetical protein | AAEL015259 | UNK | 1.16 |
| hypothetical protein | AAEL015613 | UNK | -0.94 |
| conserved hypothetical protein | AAEL015634 | UNK | 1.44 |
