# Supplementary material for: The Aedes aegypti Toll Pathway Controls Dengue Virus Infection
Source: PLoS Pathog. 2008 Jul 4;4(7):e1000098. doi: 10.1371/journal.ppat.1000098 (PMC2435278; doi:10.1371/journal.ppat.1000098)
Supplement: Table S2 — The functional groups of the total 63 genes that were regulated by DENV-2 infection in the mosquito midgut at ten days after an infected blood meal, compared to that of non-infected blood fed control mosquitoes. Functional group abbreviations: IMM, immunity; RED/STE, redox and oxidoreductive stress; CSR, chemosensory reception; DIG, blood and sugar food digestive; PROT, proteolysis; CYT/STR, cytoskeletal and structural; TRP, transport; R/T/T, replication, transcription, and translation; MET, metabolism; DIV, diverse functions; UNK, unknown functions. (0.08 MB DOC) [file ppat.1000098.s003.doc]

| **Gene Name** | **Gene ID** | **Functional Group** | **Average log2 value** |
| --- | --- | --- | --- |
| myosin vi | AAEL004227 | CS | -1.01 |
| hypothetical protein | AAEL000915 | DIV | -0.83 |
| alkaline phosphatase | AAEL000931 | DIV | 1.86 |
| hypothetical protein | AAEL001471 | DIV | -1.36 |
| outer mitochondrial translocase subunit, putative | AAEL002076 | DIV | 0.95 |
| fimbrin/plastin | AAEL002539 | DIV | -0.86 |
| niemann-pick C1 | AAEL003325 | DIV | 0.85 |
| CRAL/TRIO domain-containing protein | AAEL003347 | DIV | -0.81 |
| bmp-induced factor | AAEL003861 | DIV | 1.06 |
| pdgf/vegf receptor | AAEL003928 | DIV | -0.88 |
| ornithine aminotransferase | AAEL005289 | DIV | -0.81 |
| conserved hypothetical protein | AAEL005300 | DIV | 0.99 |
| rhodopsin | AAEL005621 | DIV | 0.82 |
| allatostatin receptor | AAEL006077 | DIV | 0.91 |
| conserved hypothetical protein | AAEL007592 | DIV | 0.93 |
| conserved hypothetical protein | AAEL008723 | DIV | -0.88 |
| conserved hypothetical protein | AAEL009257 | DIV | 0.85 |
| outer mitochondrial translocase subunit, putative | AAEL009590 | DIV | 0.95 |
| hypothetical protein | AAEL010818 | DIV | -0.82 |
| nuclear transcription factor 4 homolog isoform-C (HNF4C) | AAEL011327 | DIV | 1.02 |
| NFkappaB essential modulator, putative | AAEL012510 | DIV | -1.04 |
| conserved hypothetical protein | AAEL013912 | DIV | 0.85 |
| cell division cycle 20 (cdc20) (fizzy) | AAEL014025 | DIV | 0.81 |
| LAP4 protein, putative (Scribble protein, putative) | AAEL014906 | DIV | -0.90 |
| conserved hypothetical protein | AAEL015466 | DIV | -1.13 |
| BGBP | AAEL000652 | IMM | 0.93 |
| leucine-rich transmembrane protein | AAEL002295 | IMM | -0.85 |
| peroxiredoxin 6, prx-6 | AAEL002309 | IMM | -1.49 |
| SRPN | AAEL002715 | IMM | 1.24 |
| TOLL | AAEL003507 | IMM | 0.95 |
| conserved hypothetical protein | AAEL003841 | IMM | 1.08 |
| conserved hypothetical protein | AAEL003857 | IMM | 1.08 |
| antibacterial peptide, putative | AAEL004223 | IMM | 0.81 |
| CLIP | AAEL005093 | IMM | -0.91 |
| crk | AAEL006523 | IMM | -0.91 |
| FREP | AAEL006699 | IMM | -1.13 |
| fibrinogen and fibronectin | AAEL006704 | IMM | -0.90 |
| Niemann-Pick Type C-2, putative | AAEL006854 | IMM | 1.14 |
| SRPN | AAEL007765 | IMM | -0.96 |
| CASP | AAEL014658 | IMM | -0.90 |
| lysozyme P, putative | AAEL015404 | IMM | 0.94 |
| HOP | AEG_V1.17123 | IMM | -1.04 |
| CAT | AEG_V1.18215 | IMM | -0.85 |
| LRP1 | AEG_V1.19881 | IMM | 0.83 |
| HSC70-3 | AEG_V1.21321 | IMM | -0.89 |
| carbonic anhydrase | AAEL004930 | MET | -0.86 |
| CASP | AAEL011562 | MET | -0.84 |
| aminoacylase, putative | AAEL011206 | PROT | 0.89 |
| carboxylesterase | AAEL000898 | RED/STE | -0.85 |
| cytochrome P450 | AAEL001960 | RED/STE | -0.86 |
| cytochrome P450 | AAEL003399 | RED/STE | 0.85 |
| cytochrome P450 | AAEL006798 | RED/STE | -0.96 |
| cytochrome P450 | AAEL009117 | RED/STE | -0.99 |
| ribosomal protein S6 | AAEL000032 | R/T/T | -0.93 |
| eukaryotic translation initiation factor 3, theta subunit | AAEL007078 | R/T/T | -1.19 |
| conserved hypothetical protein | AAEL014189 | R/T/T | 0.86 |
| aquaporin | AAEL003550 | TRP | -0.80 |
| succinate dehydrogenase | AAEL010608 | TRP | -0.87 |
| conserved hypothetical protein | AAEL002263 | UKN | 1.14 |
| conserved hypothetical protein | AAEL004522 | UKN | 1.12 |
| hypothetical protein | AAEL005236 | UKN | -1.90 |
| conserved hypothetical protein | AAEL009533 | UKN | 1.20 |
| conserved hypothetical protein | AAEL012295 | UKN | -0.83 |
