# Supplementary material for: The Aedes aegypti Toll Pathway Controls Dengue Virus Infection
Source: PLoS Pathog. 2008 Jul 4;4(7):e1000098. doi: 10.1371/journal.ppat.1000098 (PMC2435278; doi:10.1371/journal.ppat.1000098)
Supplement: Table S3 — Averaged data from three biological replicate real time qPCR assays of the expression of defensin, cecropin, Cactus, and Rel1in Cactus, and Cactus & Rel1 depleted mosquitoes (A) and in Caspar, and Caspar & Rel2 depleted mosquitoes (B). C. Fold change in the expression of selected immune genes in aseptic mosquitoes compared to septic mosquitoes. S.E., standard error. (0.06 MB DOC) [file ppat.1000098.s004.doc]

A.

|  |  | **dsCactus RNAi** | | **dsCactus + Rel1 RNAi** | |
| --- | --- | --- | --- | --- | --- |
| **Gene name** | **Transcript ID** | **Fold** | **S. E.** | **Fold** | **S. E.** |
| DEFC | AAEL003832-RA | 1.91 | 0.27 | 1.31 | 0.58 |
| CECG | AAEL015515-RA | 2.48 | 0.98 | 1.89 | 0.76 |
| Cactus | AAEL000709-RA | -1.23 | 0.24 | -3.07 | 0.29 |
| REL1 | AAEL007696-RA | 1.77 | 0.31 | -1.12 | 0.10 |

B.

|  |  | **dsCaspar RNAi** | | **dsCaspar + Rel2 RNAi** | |
| --- | --- | --- | --- | --- | --- |
| **Gene name** | **Transcript ID** | **Fold** | **S. E.** | **Fold** | **S. E.** |
| DEF | AAEL003832-RA | 1.35 | 0.36 | -11.44 | 1.10 |
| CEC | AAEL015515-RA | 2.84 | 0.55 | -8.28 | 3.12 |
| Caspar | AAEL003579-RA | -1.52 | 0.39 | 1.00 | 0.13 |
| REL2 | AAEL007624-RA | -1.37 | 0.54 | -1.10 | 0.14 |

C.

| **Gene name** | **Transcript ID** | **Fold** | **S. E.** |
| --- | --- | --- | --- |
| Attacin | AAEL003389-RA | -7.30 | 3.06 |
| CECG | AAEL015515-RA | -4.28 | 1.34 |
| DEFC | AAEL003832-RA | -2.01 | 0.26 |
| Diptericin | AAEL004833-RA | 1.28 | 0.08 |
| Gambicin | AAEL004522-RA | -1.75 | 0.30 |
| Caspar | AAEL003579-RA | 1.35 | 0.17 |
| Cactus | AAEL000709-RA | 1.50 | 0.43 |
| GNBPB1 | AAEL003889-RA | 1.45 | 0.15 |
| PGRPLC | AAEL014640-RA | 1.28 | 0.05 |
| Rel1 | AAEL007696-RA | 1.08 | 0.01 |
| Rel2 | AAEL007624-RA | 1.43 | 0.01 |
