# Supplementary material for: The Aedes aegypti Toll Pathway Controls Dengue Virus Infection
Source: PLoS Pathog. 2008 Jul 4;4(7):e1000098. doi: 10.1371/journal.ppat.1000098 (PMC2435278; doi:10.1371/journal.ppat.1000098)
Supplement: Table S4 — A. Averaged data from three independent biological replicate plaque assays of the virus titer in the midguts of the Cactus, Caspar, MYD88 and GFP dsRNA treated mosquitoes. B. Results from three independent biological replicate plaque assays of the virus titer in the midgut of antibiotic treated aseptic and non-treated septic mosquitoes. S.E., standard error; S, significant; NS, Non-significant. (0.04 MB DOC) [file ppat.1000098.s005.doc]

A.

| **Treatment** | **Midgut #** | **Mean PFU ± S.E., x 10 4/ midgut** | **P value** | **Significance** |
| --- | --- | --- | --- | --- |
| dsGFP | 35 | 5.03 ± 0.01 | ─ | ─ |
| dsCact | 15 | 1.34 ± 0.00 | 0.018 | S |
| dsCasp | 15 | 7.35± 1.09 | 0.14 | NS |
| dsMyD88 | 15 | 13.45± 0.93 | 0.0003 | S |

B.

| **Treatment** | **Midgut #** | **Mean PFU ± S.E., x 10 4/ midgut** | **P value** | **Significance** |
| --- | --- | --- | --- | --- |
| Septic | 20 | 3.17 ± 0.46 | ─ | ─ |
| Aseptic | 25 | 6.38 ± 1.26 | 0.034 | S |
