# Supplementary material for: The Aedes aegypti Toll Pathway Controls Dengue Virus Infection
Source: PLoS Pathog. 2008 Jul 4;4(7):e1000098. doi: 10.1371/journal.ppat.1000098 (PMC2435278; doi:10.1371/journal.ppat.1000098)
Supplement: Table S5 — The prime sequences used for the real-time qPCR assays. (0.04 MB DOC) [file ppat.1000098.s006.doc]

| **Transcript ID** | **Primer name** | **Sequence** |
| --- | --- | --- |
| AAEL007696-RA | Rel1A Forward | 5’-TGGTGGTGGTGTCCTGCGTAAC-3’ |
|  | Reverse | 5’-CTGCCTGGCGTGACCGTATCC-3’ |
| AAEL004522-RA | GAM Forward | 5’-GCCAAAACCTGTTCCTCTTG-3’ |
|  | Reverse | 5’-CGATGTAGCATTCGGTGATG-3’ |
| AAEL003832-RA | DEFC Forward | 5’-TTGTTTGCTTCGTTGCTCTTT-3’ |
|  | Reverse | 5’-ATCTCCTACACCGAACCCACT-3’ |
| AAEL015515-RA | CECG Forward | 5’-TCACAAAGTTATTTCTCCTGATCG-3’ |
|  | Reverse | 5’-GCTTTAGCCCCAGCTACAAC-3’ |
| AAEL000709-RA | Cactus Forward | 5’-AGACAGCCGCACCTTCGATTCC-3’ |
|  | Reverse | 5’-CGCTTCGGTAGCCTCGTGGATC-3’ |
| AAEL003889-RA | GNBPB1Forward | 5’-GAAGAATACAGCAAGGGGTTC-3’ |
|  | Reverse | 5’-TGCCAGATTCAGGATAAAGTG-3’ |
| AAEL003389-RA | Attacin Forward | 5’-TTGGCAGGCACGGAATGTCTTG-3’ |
|  | Reverse | 5’-TGTTGTCGGGACCGGGAAGTG-3’ |
| AAEL004833-RA | Diptericin Forward | 5’-ATCCGATTCAGAATTCGCTTT-3’ |
|  | Reverse | 5’-TTTACCGTCTCCCTGAAATCC-3’ |
| AAEL014640-RA | PGRPLC Forward | 5’-CTTCCAGCCCTTCATCGTCCAC-3’ |
|  | Reverse | 5’-CGCATTCGCTGTCACTGGTCTC-3’ |
| AAEL007624-RA | Rel2 Forward | 5’-GCTCAGTGCTACCGTGGGAAAC-3’ |
|  | Reverse | 5’-CGGGTTCGCTCTGGCATTTGTC-3’ |
| AAEL003579-RA | Caspar Forward | 5’-GAATCCGAGCGAGCCGATGC-3’ |
|  | Reverse | 5’-CGTAGTCCAGCGTTGTGAGGTC-3’ |
| AAEL009496-RA | S7 Forward | 5’-GGGACAAATCGGCCAGGCTATC-3’ |
|  | Reverse | 5’-TCGTGGACGCTTCTGCTTGTTG-3’ |
