# Supplementary material for: The Aedes aegypti Toll Pathway Controls Dengue Virus Infection
Source: PLoS Pathog. 2008 Jul 4;4(7):e1000098. doi: 10.1371/journal.ppat.1000098 (PMC2435278; doi:10.1371/journal.ppat.1000098)
Supplement: Table S6 — The expression data of all the genes that are shown in the hierarchical cluster matrix (Fig. 3C). (0.30 MB DOC) [file ppat.1000098.s007.doc]

| **Gene Name** | **Gene ID** | **dsCaspar** | **dsCactus** | **IC** | **IM** | **Number in Fig. 3C** |
| --- | --- | --- | --- | --- | --- | --- |
| cell division cycle 20 (cdc20) (fizzy) | AAEL014025 | 0.03 | -0.09 | -0.97 | 0.81 |  |
| conserved hypothetical protein | AAEL013912 | 0.06 | -0.08 | -0.88 | 0.85 |  |
| conserved hypothetical protein | AAEL005300 | 0.06 | 0.05 | -0.94 | 0.99 |  |
| niemann-pick C1 | AAEL003325 | 0.08 | 0.05 | -0.95 | 0.85 | 34 |
| alkaline phosphatase | AAEL000931 | -0.38 | 1.31 | -1.39 | 1.86 |  |
| hypothetical protein | AAEL012085 | 0.81 | 0.36 | -0.91 | 0.24 |  |
| leucine-rich transmembrane protein | AAEL011734 | 0.84 | -1.36 | -0.25 | -0.12 |  |
| conserved hypothetical protein | AAEL006406 | 1.84 | -1.72 | 0.17 | 0.20 |  |
| conserved hypothetical protein | AAEL004522 | 0.85 | -1.41 | 0.85 | 1.12 | 10 |
| conserved hypothetical protein | AAEL003849 | 1.70 | -0.81 | 0.82 | 0.05 | 7 |
| conserved hypothetical protein | AAEL008320 | 0.17 | -2.06 | 1.02 | 0.12 |  |
| hypothetical protein | AAEL006323 | -0.05 | -1.98 | 1.11 | 0.21 |  |
| modifier of mdg4 | AAEL010576 | 0.00 | -1.03 | 1.18 | -0.04 |  |
| conserved hypothetical protein | AAEL006087 | -0.02 | -1.01 | 1.09 | 0.00 |  |
| tricarboxylate transport protein | AAEL005991 | 0.09 | -0.96 | 1.24 | 0.19 |  |
| NADH dehydrogenase, putative | AAEL010673 | -0.16 | -0.93 | 0.89 | 0.14 |  |
| caspase-1 | AAEL003439 | -0.09 | -0.82 | 0.80 | 0.02 | 1 |
| conserved hypothetical protein | AAEL009861 | -0.28 | -1.52 | 1.11 | 0.18 |  |
| mitochondrial ribosomal protein, S18C, putative | AAEL001615 | 0.05 | -0.97 | 0.89 | 0.44 |  |
| mediator complex, subunit, putative | AAEL005934 | -0.06 | -1.07 | 1.02 | 0.39 |  |
| molybdopterin biosynthesis moeb protein | AAEL004607 | -0.06 | -1.06 | 1.08 | 0.34 |  |
| conserved hypothetical protein | AAEL006502 | -0.23 | -0.98 | 1.28 | 0.38 |  |
| aminoacylase, putative | AAEL011206 | -0.14 | -1.00 | 0.23 | 0.89 |  |
| outer mitochondrial translocase subunit, putative | AAEL009590 | -0.30 | -1.08 | 0.71 | 0.95 |  |
| outer mitochondrial translocase subunit, putative | AAEL002076 | -0.30 | -1.08 | 0.71 | 0.95 |  |
| conserved hypothetical protein | AAEL002263 | -1.18 | -1.22 | 0.72 | 1.14 |  |
| alkaline phosphatase | AAEL003313 | -0.82 | -1.64 | 0.00 | 0.13 |  |
| conserved hypothetical protein | AAEL003178 | -0.86 | -1.53 | -0.09 | -0.18 |  |
| short-chain dehydrogenase | AAEL006224 | -0.93 | -1.31 | -0.17 | -0.01 |  |
| serine protease | AAEL002124 | -0.82 | -1.15 | -0.12 | 0.05 |  |
| conserved hypothetical protein | AAEL011400 | -1.02 | -1.00 | 0.58 | -0.08 |  |
| conserved hypothetical protein | AAEL013775 | -1.22 | -1.26 | 0.62 | 0.06 |  |
| myosin light chain 1, | AAEL012207 | -0.84 | -0.91 | 0.41 | 0.02 |  |
| tyrosyl-dna phosphodiesterase | AAEL015141 | -0.82 | -0.97 | 0.33 | 0.03 |  |
| tyrosyl-dna phosphodiesterase | AAEL011629 | -0.82 | -0.97 | 0.33 | 0.03 |  |
| conserved hypothetical protein | AAEL012073 | -0.83 | -0.90 | 0.19 | -0.02 |  |
| hypothetical protein | AAEL003153 | -0.92 | -1.16 | 0.24 | -0.49 |  |
| conserved hypothetical protein | AAEL005358 | -1.34 | -1.08 | -0.32 | 0.08 |  |
| Inhibitor of nuclear factor kappa B kinase beta subunit | AAEL003245 | -1.24 | -0.96 | -0.43 | -0.04 |  |
| glycogenin | AAEL014863 | -0.85 | -0.89 | 0.07 | 0.13 |  |
| protein phosphatase 2c | AAEL007171 | -1.10 | -0.96 | 0.00 | 0.16 |  |
| conserved hypothetical protein | AAEL013774 | -1.25 | -1.02 | -0.02 | -0.24 |  |
| hypothetical protein | AAEL001392 | -0.91 | -0.96 | -0.76 | -0.29 |  |
| conserved hypothetical protein | AAEL004976 | 0.04 | -2.46 | -1.57 | 0.10 |  |
| peroxinectin | AAEL004386 | 0.05 | -1.75 | -1.11 | -0.03 | 18 |
| mfs transporter | AAEL012183 | -0.01 | -1.32 | -0.95 | 0.03 |  |
| superoxide dismutase | AAEL006271 | 0.00 | -1.10 | -0.84 | -0.01 | 22 |
| hypothetical protein | AAEL012504 | -0.19 | -1.13 | -0.82 | -0.02 |  |
| serine/threonine protein kinase | AAEL000217 | -0.05 | -0.87 | -0.89 | -0.06 |  |
| regulator of chromosome condensation | AAEL007799 | -0.08 | -0.99 | -0.89 | -0.03 |  |
| deoxyuridine 5'-triphosphate nucleotidohydrolase | AAEL007323 | -0.04 | -1.53 | -1.47 | 0.04 |  |
| yellow protein precursor, putative | AAEL014001 | 0.05 | -1.20 | -0.97 | 0.02 |  |
| peroxinectin | AAEL004388 | 0.09 | -2.05 | -1.69 | 0.05 | 19 |
| dopachrome-conversion enzyme (DCE) isoenzyme, putative | AAEL005325 | 0.04 | -1.20 | -1.05 | -0.05 |  |
| superoxide dismutase | AAEL011498 | 0.08 | -1.21 | -0.90 | -0.16 | 24 |
| superoxide dismutase | AAEL000274 | 0.08 | -1.18 | -0.91 | -0.12 | 21 |
| diacylglycerol o-acyltransferase | AAEL008878 | 0.11 | -1.12 | -0.95 | -0.20 |  |
| conserved hypothetical protein | AAEL012865 | -0.34 | -1.62 | -0.97 | -0.44 |  |
| histone H3 | AAEL003685 | 0.34 | -2.54 | -1.07 | 0.22 |  |
| hypothetical protein | AAEL002473 | 0.39 | -2.37 | -1.10 | 0.05 |  |
| conserved hypothetical protein | AAEL000776 | -0.07 | -2.62 | -1.19 | 0.07 |  |
| conserved hypothetical protein | AAEL015080 | 0.13 | -1.48 | -0.90 | 0.08 |  |
| hypothetical protein | AAEL009962 | 0.30 | -2.58 | -1.43 | 0.08 |  |
| amino acid transporter | AAEL007458 | 0.25 | -2.79 | -1.40 | -0.08 |  |
| geminin, putative | AAEL009773 | 0.35 | -1.01 | -0.95 | -0.05 |  |
| histone H4 | AAEL003673 | 0.38 | -1.54 | -1.32 | -0.02 |  |
| peroxinectin | AAEL004390 | 0.27 | -1.36 | -1.03 | 0.06 | 20 |
| histone h2a | AAEL003862 | 0.58 | -1.59 | -1.34 | 0.19 |  |
| histone h2a | AAEL003820 | 0.54 | -1.53 | -0.94 | 0.19 |  |
| histone h2a | AAEL003818 | 0.54 | -1.53 | -0.94 | 0.19 |  |
| histone h2a | AAEL003851 | 0.54 | -1.53 | -0.94 | 0.19 |  |
| histone h2a | AAEL003826 | 0.54 | -1.53 | -0.94 | 0.19 |  |
| 4-nitrophenylphosphatase | AAEL007097 | 0.60 | -1.30 | -1.53 | 0.02 |  |
| amidophosphoribosyltransferase | AAEL003581 | 0.18 | -0.89 | -0.99 | -0.17 |  |
| Thymidylate kinase, putative | AAEL001246 | -0.12 | -0.94 | -1.05 | -0.08 |  |
| phosphoenolpyruvate carboxykinase | AAEL000006 | -0.15 | -0.92 | -1.11 | -0.01 |  |
| dead box atp-dependent rna helicase | AAEL008500 | -0.47 | -1.30 | -1.50 | -0.11 |  |
| peroxiredoxins, prx-1, prx-2, prx-3 | AAEL004112 | 0.06 | -0.84 | -1.43 | -0.27 | 25 |
| heat shock protein | AAEL014845 | -0.07 | -0.90 | -0.81 | -0.52 |  |
| hypothetical protein | AAEL011141 | 0.01 | -0.87 | -0.82 | -0.46 |  |
| conserved hypothetical protein | AAEL004699 | 0.03 | -0.98 | -0.84 | -0.44 |  |
| purine biosynthesis protein 6, pur6 | AAEL003606 | 0.04 | -1.30 | -0.85 | -0.50 |  |
| heat shock protein | AAEL014843 | 0.00 | -0.84 | -1.04 | -0.37 |  |
| abc transporter | AAEL008138 | -0.08 | -0.94 | -0.84 | -0.29 |  |
| serine hydroxymethyltransferase | AAEL002510 | -0.25 | -1.01 | -0.97 | -0.68 |  |
| fibrinogen and fibronectin | AAEL006704 | 0.31 | -1.13 | 0.07 | -0.90 | 33 |
| aquaporin | AAEL003550 | -0.04 | -0.85 | -0.03 | -0.80 |  |
| NFkappaB essential modulator, putative | AAEL012510 | -0.03 | 0.04 | -0.91 | -1.04 | 12 |
| hypothetical protein | AAEL010818 | 0.24 | -0.15 | -0.95 | -0.82 |  |
| eukaryotic translation initiation factor 3, theta subunit | AAEL007078 | 0.16 | -0.23 | -0.92 | -1.19 |  |
| HSC70-3 | Aaeg_N51900 | 0.18 | 0.96 | -1.00 | -0.89 |  |
| serine protease inhibitor, serpin | AAEL008364 | 0.17 | 1.28 | -0.95 | -0.03 | 55 |
| lumbrokinase-3(1) precursor, putative | AAEL007593 | 0.16 | 1.38 | -0.82 | 0.15 | 47 |
| serine protease | AAEL005064 | 0.32 | 1.55 | -0.85 | 0.06 | 46 |
| conserved hypothetical protein | AAEL006883 | 0.26 | 2.25 | -0.80 | -0.59 |  |
| fibrinogen and fibronectin | AAEL005194 | 0.87 | 1.15 | -0.24 | 0.02 |  |
| proacrosin, putative | AAEL014139 | 0.84 | 2.34 | -0.03 | 0.03 |  |
| galactose-specific C-type lectin, putative | AAEL014382 | 0.89 | 1.76 | 0.29 | 0.02 |  |
| galactose-specific C-type lectin, putative | AAEL011607 | 0.90 | 1.65 | 0.08 | -0.13 |  |
| serine protease inhibitor 4, serpin-4 | AAEL007765 | -0.01 | 0.84 | 0.17 | -0.96 | 61 |
| conserved hypothetical protein | AAEL003857 | -1.96 | 1.00 | -0.13 | 1.08 | 8 |
| conserved hypothetical protein | AAEL003841 | -2.16 | 0.97 | 0.17 | 1.08 |  |
| antibacterial peptide, putative | AAEL015515 | -2.99 | 1.39 | 1.05 | 0.13 | 6 |
| macroglobulin/complement | AAEL001802 | -1.23 | 1.70 | 0.23 | 0.06 |  |
| serine protease | AAEL000074 | -1.53 | 1.26 | -0.01 | 0.17 |  |
| dopachrome-conversion enzyme (DCE), putative | AAEL000024 | -0.82 | 0.85 | -0.09 | 0.12 |  |
| conserved hypothetical protein | AAEL013840 | -0.83 | 1.20 | -0.27 | -0.28 |  |
| conserved hypothetical protein | AAEL003832 | -1.67 | 0.95 | -1.81 | 0.14 | 9 |
| niemann-pick C1 | AAEL009531 | -0.83 | 0.03 | 1.43 | -0.07 | 35 |
| gram-negative bacteria binding protein | AAEL007064 | -1.08 | 0.12 | 0.89 | 0.09 | 29 |
| LRP1 | Aaeg_N19019 | 0.07 | 0.00 | 1.07 | 0.83 |  |
| gram-negative bacterial binding protein, putative | AAEL000652 | -0.03 | 0.04 | 0.81 | 0.93 | 28 |
| macroglobulin/complement | AAEL012267 | 0.80 | 0.11 | 1.33 | 0.08 | 41 |
| serine protease | AAEL002595 | 0.36 | 1.62 | 0.94 | 0.12 |  |
| macroglobulin/complement | AAEL001794 | 0.31 | 1.52 | 0.90 | 0.19 | 40 |
| clip-domain serine protease, putative | AAEL003253 | 0.00 | 1.64 | 1.04 | 0.21 | 45 |
| serine protease | AAEL002629 | 0.14 | 1.81 | 1.12 | 0.15 |  |
| tep2 | AAEL014755 | 0.17 | 1.63 | 1.19 | -0.02 | 42 |
| yellow protein precursor | AAEL005738 | 0.13 | 1.38 | 0.85 | -0.03 |  |
| galactose-specific C-type lectin, putative | AAEL011455 | 0.22 | 2.47 | 1.10 | 0.13 | 50 |
| serine protease | AAEL002585 | 0.22 | 2.29 | 0.83 | 0.04 |  |
| protein tyrosine phosphatase, putative | AAEL012471 | -0.87 | 1.56 | 1.08 | -0.06 | 16 |
| serine protease inhibitor, serpin | AAEL013936 | 0.16 | 1.43 | 1.35 | 0.04 | 57 |
| macroglobulin/complement | AAEL000087 | 0.32 | 1.90 | 1.82 | 0.08 | 39 |
| embryonic polarity dorsal | AAEL007696 | 0.16 | 1.01 | 0.92 | -0.10 | 64 |
| serine protease inhibitor, serpin | AAEL014079 | -0.03 | 1.00 | 0.92 | -0.02 | 59 |
| galactose-specific C-type lectin, putative | AAEL014390 | 0.19 | 0.94 | 0.99 | 0.16 | 52 |
| galactose-specific C-type lectin, putative | AAEL011619 | 0.28 | 1.13 | 0.99 | 0.09 | 51 |
| hypothetical protein | AAEL013934 | 0.26 | 0.91 | 1.34 | 0.22 | 56 |
| organic cation transporter | AAEL009206 | 0.29 | 0.94 | 1.68 | 0.15 |  |
| lysozyme P, putative | AAEL015404 | 0.08 | 1.11 | 1.01 | 0.94 | 11 |
