# Supplementary material for: The Aedes aegypti Toll Pathway Controls Dengue Virus Infection
Source: PLoS Pathog. 2008 Jul 4;4(7):e1000098. doi: 10.1371/journal.ppat.1000098 (PMC2435278; doi:10.1371/journal.ppat.1000098)
Supplement: Text S1 — This section refers to other dengue infection responsive genes. (0.05 MB DOC) [file ppat.1000098.s008.doc]

**Non-immune genes that were differentially expressed in response to dengue infection**

Oxidoreductive and stress responses to dengue infection

A variety of genes implicated in oxidoreductive and stress responsive processes displayed dengue infection-responsive regulation: Ten were down-regulated and five were up-regulated. It is particularly interesting that several differentially expressed genes with known involvement in oxidative defense were repressed, including three members of each of the heme peroxidase (HPX) and superoxide dismutase (SOD) families and two members of the thioredoxins (TPXs). Several other proteins related to oxidoreductive processes were up-regulated by virus infection; these included one NADH dehydrogenase (AAEL010673) and two mitochondrial ribosomal proteins (AAEL005413 and AAEL001615). The expression of five heat shock proteins was suppressed by the virus infection, while one (AAEL003215) was up-regulated. Dengue virus has been suggested to use the heat shock protein 70 and 90 (HSP70 and 90) family proteins as cell receptors for entry [1], and the Onyong-nyong virus has been shown in *An. gambiae* to induce the expression of the heat shock protein cognate 70B (HSC70B), which has an inhibitory effect on the replication of this virus [2]. These expression signatures reflect the fact that the dengue infection is imposing stress on the mosquito, while at the same time the virus seems to be suppressing the oxidative stress responsive system.

Responses of other physiological systems to dengue infection

Forty-four genes with functional roles in replication, transcription, and translation were regulated by dengue infection, with the majority (88.6%) of these being suppressed. Several histones showed the most dramatic down-regulation, with 23 of 39 being suppressed, including histones 2a, 3, and 4. This down-regulation is likely to represent a defense mechanism, since the virus requires these conserved host proteins for their replication. Previous studies of a simian virus-40 (SV40) have shown that histone synthesis is coordinated with viral DNA replication [3]. Inhibition of transcription and translation has also been observed in Sindbis virus-infected mammalian cells [4,5]**.**

In the carcass tissue, 21 of 25 genes with putative metabolic functions were down-regulated, while the remaining four were up-regulated. Virus infection has been shown to affect host metabolic networks in for instance the case of human cytomegalovirus (HCMV) infection, which leads to an increase in the levels of metabolites involved in glycolysis, the citric acid cycle, and pyrimidine [6]. Several other genes, including glycerol-phosphate, were down-regulated in their assays. Here, we also observed down-regulation of a glycolytic upstream component, glycerol kinase (AAEL006239), and the glycine-rich RNA binding protein (AAEL015143). This major down-regulation may be a result of a general inhibition of transcription and translation or may indicate a specific inhibition of certain metabolic functions during virus infection.

Twenty genes with predicted roles in transport processes were regulated by dengue infection: The nine induced genes included an organic cation transporter, a tricarboxylate transport, a sodium/sialic acid co-transporter, an ion channel Nompc, and a neurotransmitter gated ion channel. Sindbis virus has also been reported to induce transport processes in *Ae. aegypti [5]*, and this effect was thought to provide a mechanism for influencing the vesicle transport; alternatively, some plasma membrane transporters may act as receptors for the virus [5]. Another 156 genes that were differentially expressed in the carcass belong to diverse functional groups, and 73 have unknown functions. Interesting examples of these genes include two transcripts that have been implicated in reproduction (AAEL005381 and AAEL010585), three transcripts involved in developmental processes (AAEL007397, AAEL006832 and AAEL003650), and an odorant-binding protein gene (AAEL010410). Evidence has suggested that the dengue envelope protein can interact with the small ubiquitin-like modifier-1 conjugating enzyme 9 [7], and an ubiquitin-conjugating enzyme H (AAEL013633) was observed to be up-regulated in the infected carcass.

**References**

1. Reyes-Del Valle J, Chavez-Salinas S, Medina F, Del Angel RM (2005) Heat shock protein 90 and heat shock protein 70 are components of dengue virus receptor complex in human cells. J Virol 79: 4557-4567.

2. Sim C, Hong YS, Tsetsarkin KA, Vanlandingham DL, Higgs S, et al. (2007) *Anopheles gambiae* heat shock protein cognate 70B impedes *o'nyong-nyong* virus replication. BMC Genomics 8: 231.

3. Tan KB (1977) Histones: metabolism in simian virus 40-infected cells and incorporation into virions. Proc Natl Acad Sci U S A 74: 2805-2809.

4. Gorchakov R, Frolova E, Frolov I (2005) Inhibition of transcription and translation in Sindbis virus-infected cells. J Virol 79: 9397-9409.

5. Sanders HR, Foy BD, Evans AM, Ross LS, Beaty BJ, et al. (2005) Sindbis virus induces transport processes and alters expression of innate immunity pathway genes in the midgut of the disease vector, *Aedes aegypti*. Insect Biochem Mol Biol 35: 1293-1307.

6. Munger J, Bajad SU, Coller HA, Shenk T, Rabinowitz JD (2006) Dynamics of the cellular metabolome during human cytomegalovirus infection. PLoS Pathog 2: e132.

7. Chiu MW, Shih HM, Yang TH, Yang YL (2007) The type 2 dengue virus envelope protein interacts with small ubiquitin-like modifier-1 (SUMO-1) conjugating enzyme 9 (Ubc9). J Biomed Sci 14: 429-444.
